# Accurate assembly of the olive baboon (*Papio anubis*) genome using long-read and Hi-C data

**DOI:** 10.1093/gigascience/giaa134

**Published:** 2020-12-07

**Authors:** Sanjit Singh Batra, Michal Levy-Sakin, Jacqueline Robinson, Joseph Guillory, Steffen Durinck, Tauras P Vilgalys, Pui-Yan Kwok, Laura A Cox, Somasekar Seshagiri, Yun S Song, Jeffrey D Wall

**Affiliations:** Computer Science Division, University of California Berkeley, 2626 Hearst Avenue, Berkeley, CA 94720, USA; Cardiovascular Research Institute, University of California San Francisco, 555 Mission Bay Boulevard South, San Francisco, CA 94158, USA; Institute for Human Genetics, University of California San Francisco, 513 Parnassus Avenue, San Francisco, CA 94143, USA; Department of Molecular Biology, Genentech, Inc., 1 DNA Way, South San Francisco, CA 94080, USA; Department of Molecular Biology, Genentech, Inc., 1 DNA Way, South San Francisco, CA 94080, USA; Bioinformatics and Computational Biology Department, Genentech, Inc., 1 DNA Way, South San Francisco, CA 94080, USA; Department of Evolutionary Anthropology, Duke University, 130 Science Drive, Durham, NC 27705, USA; Cardiovascular Research Institute, University of California San Francisco, 555 Mission Bay Boulevard South, San Francisco, CA 94158, USA; Institute for Human Genetics, University of California San Francisco, 513 Parnassus Avenue, San Francisco, CA 94143, USA; Center for Precision Medicine, Department of Internal Medicine, Section of Molecular Medicine, Wake Forest School of Medicine, 475 Vine Drive, Winston-Salem, NC 27101, USA; Southwest National Primate Research Center, Texas Biomedical Research Institute, 8715 W. Military Drive, San Antonio, TX 78227, USA; Department of Molecular Biology, Genentech, Inc., 1 DNA Way, South San Francisco, CA 94080, USA; Computer Science Division, University of California Berkeley, 2626 Hearst Avenue, Berkeley, CA 94720, USA; Department of Statistics, University of California Berkeley, 367 Evans Hall, Berkeley, CA 94720, USA; Chan Zuckerberg Biohub, Mission Bay, San Francisco, CA 94158, USA; Institute for Human Genetics, University of California San Francisco, 513 Parnassus Avenue, San Francisco, CA 94143, USA

## Abstract

**Background:**

Baboons are a widely used nonhuman primate model for biomedical, evolutionary, and basic genetics research. Despite this importance, the genomic resources for baboons are limited. In particular, the current baboon reference genome Panu_3.0 is a highly fragmented, reference-guided (i.e., not fully *de novo*) assembly, and its poor quality inhibits our ability to conduct downstream genomic analyses.

**Findings:**

Here we present a *de novo* genome assembly of the olive baboon (*Papio anubis*) that uses data from several recently developed single-molecule technologies. Our assembly, Panubis1.0, has an N50 contig size of ∼1.46 Mb (as opposed to 139 kb for Panu_3.0) and has single scaffolds that span each of the 20 autosomes and the X chromosome.

**Conclusions:**

We highlight multiple lines of evidence (including Bionano Genomics data, pedigree linkage information, and linkage disequilibrium data) suggesting that there are several large assembly errors in Panu_3.0, which have been corrected in Panubis1.0.

## Data Description

### Introduction

Baboons are ground-living monkeys native to Africa and the Arabian Peninsula. Owing to their relatively large size, abundance, and omnivorous diet, baboons have increasingly become a major biomedical model system (reviewed in [1]). Baboon research has been facilitated by the creation (in 1960) and maintenance of a large, pedigreed, well-phenotyped baboon colony at the Southwest National Primate Research Center (SNPRC) and an ability to control the environment of subjects in ways that are obviously not possible in human biomedical studies. For example, baboons have been used to study the effect of diet on cholesterol and triglyceride levels in experiments where all food consumption is completely controlled [2–4]. In recent years, linkage studies in baboons have helped identify genetic regions affecting a wide range of phenotypes, such as cholesterol levels [5, 6], estrogen levels [7], craniofacial measurements [8], bone density [9, 10], and lipoprotein metabolism [11]. In addition, studies have also documented that the genetic architecture of complex traits in baboons can be directly informative about analogous traits in humans (e.g., [10, 12]). In parallel, baboons have been widely used in studies of animal behavior and evolution. For example, the Amboseli Baboon Research Project has studied wild baboon troops continuously since 1971 and produced ∼300 scientific publications, including the first study of whole-genome sequence data in baboons [13].

The success of these and other studies has been mediated in part by recent advances in molecular genetics technologies. In particular, the ability to cheaply genotype and/or sequence samples of interest has led to a revolution in genetic studies of the associations between genotype and phenotype. While human genetic studies now routinely include the analyses of whole-genome sequence data from many thousands of samples (e.g., [14–18]), comparable studies in model organisms have lagged far behind. Part of the reason for this is the lack of genetic resources in non-human species. Large international projects such as the Human Genome Project [19, 20], International HapMap Project [21–23], and the 1000 Genomes Project [24–26] have provided baseline information on sequences and genetic variation, and subsequent human genetic studies have used this background information.

The first published baboon genome assembly was from a yellow baboon [13]. This assembly used a combination of Illumina paired-end and Illumina mate-pair sequence data (with mean library insert sizes ranging from 175 to 14 kb) to produce a highly fragmented assembly with contig N50 of 29 kb and scaffold N50 of 887 kb. The public olive baboon assembly, Panu_3.0, has the same problem of small contigs and scaffolds (contig N50 of 139 kb and *de novo* scaffold N50 of 586 kb) [27]. The authors of the public olive baboon assembly chose to distribute a reference-guided assembly with scaffolds mapped onto rhesus (*Macaca mulatta*) chromosomes. As a consequence, any syntenic differences between rhesus and baboon will result in large-scale assembly errors in Panu_3.0. One additional drawback of this baboon genome assembly was its informal embargo from 2008 to 2019 under the guidelines of the Fort Lauderdale agreement. Hence, its influence on scientific research has been negligible.

In this project, we focus on providing a high-quality, *de novo* genome assembly for olive baboon (*Papio anubis*, NCBI:txid9555), which we call Panubis1.0, with the hope that this resource will enable future high-resolution genotype-phenotype studies. Unlike previous baboon genome assembly efforts, we use a combination of 3 recently developed technologies (from 10x Genomics linked reads, Oxford Nanopore long reads, and Hi-C) to increase the long-range contiguity of our assembly. These newly developed technologies enable us to generate assemblies in which the autosomes (and the X chromosome) are each spanned by a single scaffold at a cost that is orders of magnitude cheaper than the Panu_3.0 assembly. We also verify that many of the large-scale syntenic differences between our Panubis1.0 and Panu_3.0 are due to errors in the public assembly rather than our own. Our assembly is available for scientific use without any restrictions.

### Genome sequencing

#### Index animal

We used individual number 15,944 (currently deceased) from the SNPRC pedigreed baboon colony for all of the sequencing and genome assembly work associated with this project.

#### 10x Genomics sequencing

High molecular weight genomic DNA extraction, sample indexing, and generation of partition barcoded libraries were performed according to the 10x Genomics (Pleasanton, CA, USA) Chromium Genome User Guide and as published previously ([28]). A mean depth of ∼60× was produced and analyzed for this project.

#### Oxford Nanopore sequencing

Libraries for the Oxford Nanopore sequencing were constructed as described previously ([29]) using DNA derived from whole blood. The sequencing was conducted at Genentech, Inc. (South San Francisco, CA, USA); we analyzed data with a mean depth of ∼15× for this project.

#### Bionano optical maps

High molecular weight DNA was extracted, nicked, and labeled using the enzyme Nt.BspQI (New England Biolabs, Ipswich, MA, USA) and imaged using the Bionano Genomics Irys system (San Diego, CA, USA) to generate single-molecule maps for assessing breaks in synteny between Panu_3.0 and Panubis1.0.

#### Hi-C sequencing

High molecular weight DNA from Jenny Tung (Duke University) was sent to Phase Genomics. We obtained ∼15× Hi-C data using previously described techniques [30].

### Genome assembly

**Figure 1:**
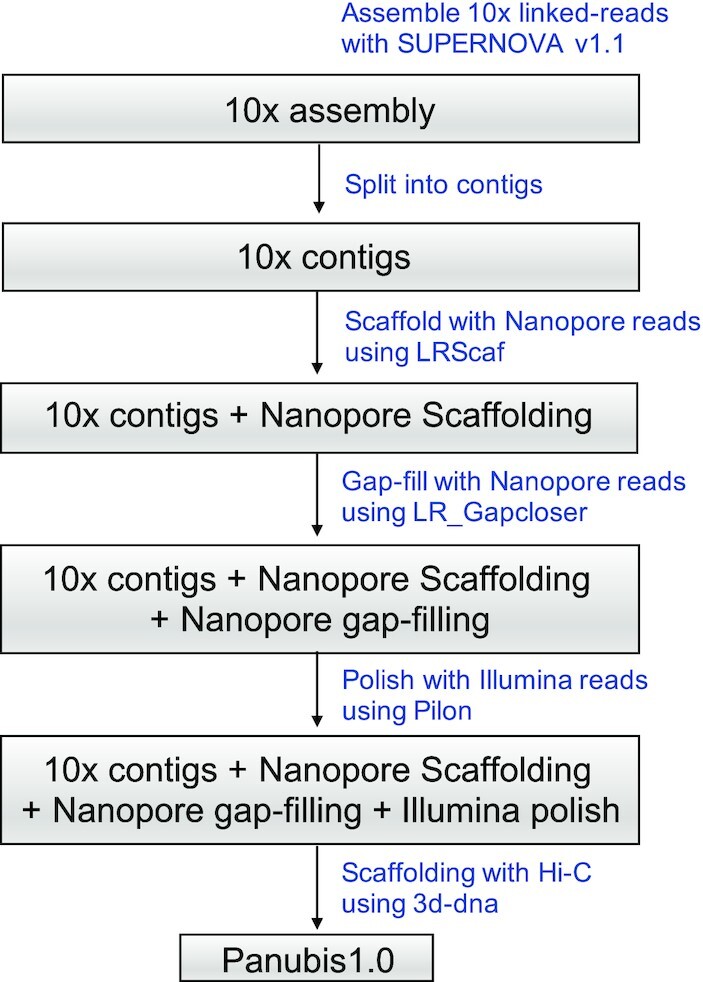
Illustration of our genome assembly strategy.

The main strength of our approach is in combining data from multiple platforms (10x Genomics linked reads, Oxford Nanopore long reads, Illumina paired-end short reads, and Hi-C), which have complementary advantages. Fig. 1 describes our assembly strategy. We began by assembling 10x Genomics reads generated with their Chromium system (mean depth ∼60×) using the Supernova assembler (Supernova assembler, RRID:SCR_016756) version 1.1, default parameters [28], which yielded an assembly with a contig N50 of ∼84 kb and a scaffold N50 of ∼15.7 Mb (Table 1). The gap lengths between the contigs in a scaffold obtained by assembling 10x linked reads are arbitrary [31]. Hence, to leverage the Oxford Nanopore long reads for gap closing, we split the 10x scaffolds at every stretch of non-zero N's to obtain a collection of contigs.

**Table 1:** Assembly statistics for each step of the adopted assembly strategy

Assembly	10x	10x Contigs	10x Contigs + Nanopore scaffolding	10x Contigs + Nanopore Scaffolding + Nanopore gap filling	10x Contigs + Nanopore scaffolding + Nanopore gap filling + Illumina polishing	Panubis1.0	Panu_3.0
Total length of scaffolds	2,892,554,220	2,809,352,255	2,871,292,557	2,871,210,925	2,870,847,162	2,869,821,163	2,959,373,024
No. of scaffolds	24,513	87,632	15,803	15,803	15,803	11,145	63,235
Scaffold N50	15,720,195	84,258	1,695,573	1,695,772	1,695,642	140,274,886	585,721
Total gap length	83,203,960	0	50,344,034	2,030,908	2,030,908	2,321,983	22,434,732
Total length of contigs	2,809,350,260	2,809,352,255	2,820,948,523	2,869,180,017	2,868,816,254	2,867,510,325	2,937,001,527
No. of contigs	87,347	87,632	62,252	17,004	17,004	15,243	122,216
Contig N50	84,258	84,258	134,222	1,469,760	1,469,602	1,455,705	138,819

Total length of scaffolds is the sum of lengths of scaffolds (including A, C, G, T and N) in each scaffold. Total gap length is the total number of N's in the assembly. Contigs are constructed by splitting the assembly at every stretch of ≥1 N. The total length of contigs is the sum of the number of sequenced base pairs (including only A, C, G, and T) in each scaffold.

We scaffolded the resulting contigs with Oxford Nanopore long reads (mean depth ∼15×) using the LR_Scaf (version 1.1.4, default parameters) [32] scaffolding method. (In accordance with the Canu assembler documentation [33], we did not have a sufficient depth of coverage to perform *de novo* assembly directly from the Nanopore reads.) This resulted in an assembly with a contig N50 of ∼134 kb and a scaffold N50 of ∼1.69 Mb (Table 1). These resulting scaffolds are more amenable to gap closing because the gap lengths (number of N's between 2 consecutive contigs) are estimated by long reads that span each gap and align to the flanking regions of that gap.

Upon performing gap closing with the same set of Oxford Nanopore long reads using LR_Gapcloser (v1.1, default parameters) [34], we obtained an assembly with a contig N50 of ∼1.47 Mb and a scaffold N50 of ∼1.69 Mb (Table 1). Note that this increase in contig N50 of ∼84 kb from the 10x Genomics linked-read assembly, to a contig N50 of ∼1.47 Mb, would not have been possible if we had simply performed gap closing with the Oxford Nanopore long reads directly on the 10x-based assembly without first splitting it into its constituent contigs. Finally, we polished the resulting assembly by aligning Illumina paired-end reads (mean depth ∼60× in PE150 reads) using Pilon (Pilon, RRID:SCR_014731) version 1.22, default parameters [35].

**Figure 2:**
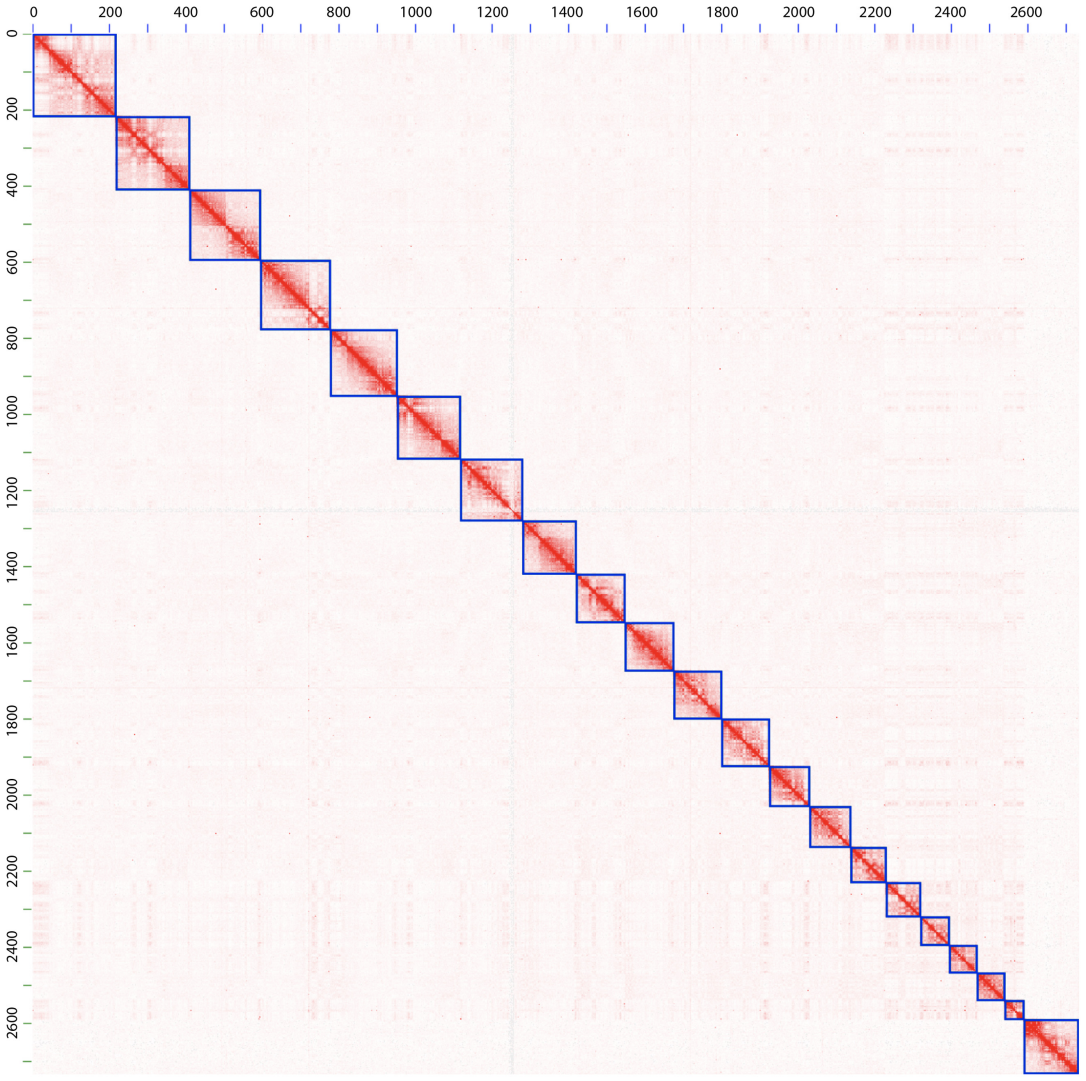
Hi-C map of our Panubis1.0 genome. The figure represents the Hi-C map obtained by aligning Hi-C paired-end reads to the Panubis1.0 genome assembly laid out on the X-axis as well as the Y-axis. Because each read-pair consists of 2 reads, a position (*i, j*) on this map represents the number of read-pairs where one read aligned to position *i* and the other read aligned to position *j* on the Panubis1.0 genome. The intensity of each pixel in this Hi-C map represents the number of reads aligning within that bin. The Hi-C map has been drawn at a resolution of 1.25 Mb. Each blue square on the diagonal represents a chromosome-length scaffold. Autosomes are listed first, ordered by size, and the last square corresponds to the X chromosome. The axes are labeled in units of megabases.

To scaffold the resulting assembly with Hi-C data, we first set aside scaffolds shorter than 50 kb, which made up only ∼1.8% of the total sequence base pairs. This was done because Hi-C–based scaffolding is more reliable for longer scaffolds because there are more Hi-C reads aligning to longer scaffolds. We then ordered and oriented the remaining scaffolds using the 3D *de novo* assembly (3d-dna) pipeline (3D de novo assembly, RRID:SCR_017227) version 180,419, default parameters [36] using ∼15× Hi-C data generated by Phase Genomics [37]. Finally, we manually corrected misassemblies in the resulting Hi-C–based assembly by visualizing the Hi-C reads aligned to the assembly, using Juicebox Assembly Tools (version 1.6.11) [38], following the strategy described in [39]. Fig. 2 shows Hi-C reads aligned to the resulting assembly, with the blue squares on the diagonal representing chromosomes.

The resulting *P. anubis* genome assembly, which we name Panubis1.0, contains ∼2.87 Gb of sequenced base pairs (non-N base pairs) and 2.3 Mb (<0.1%) of gaps (N's). Single scaffolds spanning the 20 autosomes and the X chromosome together contain 95.14% (∼2.73 Gb) of the sequenced base pairs. We number the autosomes as chr1–chr20, in decreasing order of the scaffold length, so some chromosome numbers in our convention are different from Panu_3.0’s numbering. We note that Panubis1.0 has a contig N50 of 1.46 Mb, which is a >10-fold improvement over the contig N50 (∼139 kb) of the Panu_3.0 assembly. As a result, Panubis1.0 contains 5 times fewer scaffolds (11,145 scaffolds with a scaffold N50 of ∼140 Mb) compared with the Panu_3.0 assembly (63,235 scaffolds with a scaffold N50 of ∼586 kb); see Table 1 for a further comparison of the 2 assemblies. Gene completion analysis of the assembly using BUSCO version 3 (BUSCO, RRID:SCR_015008) and the euarchontoglires odb9 ortholog dataset [40] suggests that chromosomes in the Panubis1.0 assembly contain 5,167 of 6,192 (83.4%) complete genes, comparable to 5,166 of 6,192 (83.4%) complete genes found in the chromosomes of the Panu_3.0 assembly. Furthermore, the chromosomes in the Panubis1.0 assembly contained 247 of 6,192 (4.0%) fragmented genes, comparable to 262 of 6,192 (4.2%) fragmented genes in the chromosomes of the Panu_3.0 assembly.

### Y chromosome assembly

**Figure 3:**
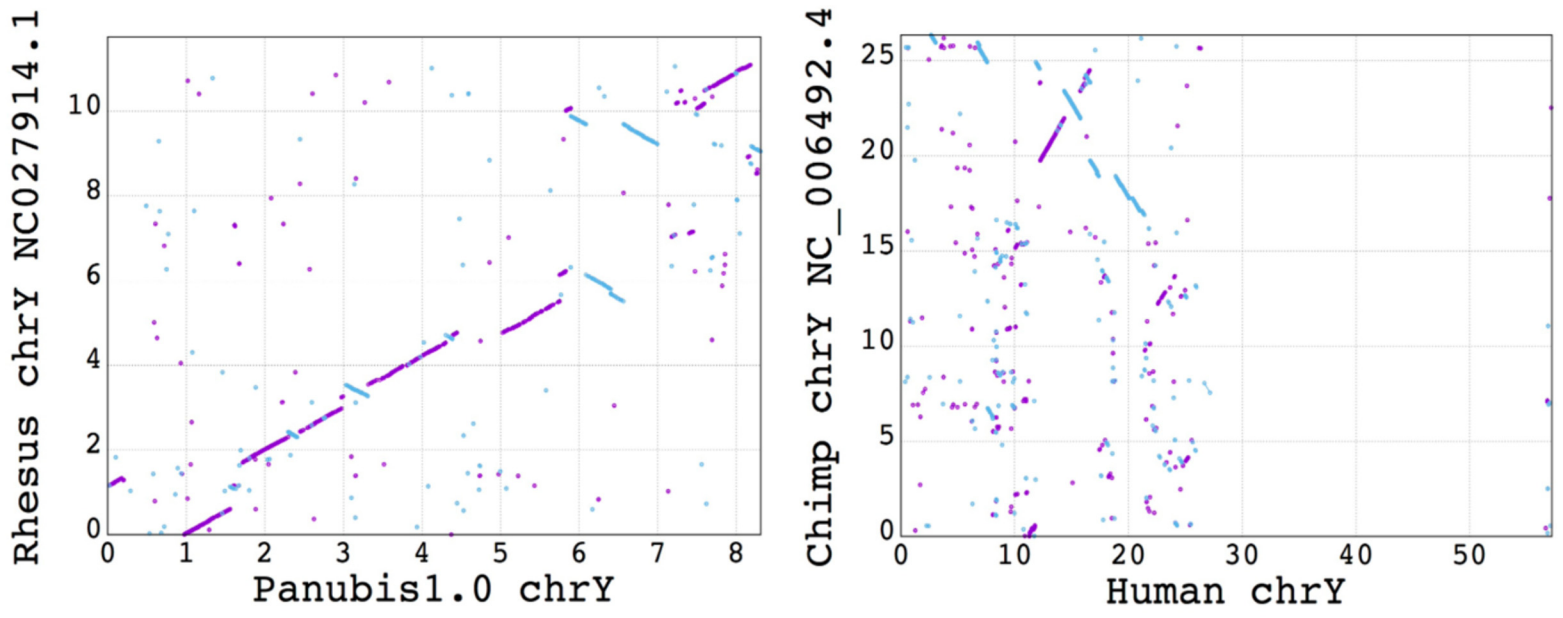
Dot plots showing chromosome Y synteny suggest that the Panubis1.0 chromosome Y is putatively at least a part of the true chromosome Y. A dot plot between rhesus chromosome Y and Panubis1.0 putative chromosome Y is shown on the left, while a dot plot between the chimpanzee chromosome Y and the human chromosome Y is shown on the right. Each dot represents an aligned block, with purple representing an alignment on the positive strand and cyan an alignment on the negative strand. The axis labels are in units of megabases. The phylogenetic distance between baboon and rhesus macaque is similar to that between human and chimpanzee. Hence, the broadly conserved synteny between the rhesus and baboon putative chromosome Y as compared to the synteny between the chimp and human chromosome Y suggests that the scaffold representing the putative chromosome Y in the Panubis1.0 assembly is indeed capturing at least a large part of chromosome Y.

The Hi-C scaffolding with 3d-dna yielded an ∼8 Mb scaffold that putatively represents part of the baboon Y chromosome. Because rhesus macaque is the phylogenetically closest species to baboons that has a chromosome-scale assembly, we aligned this putative baboon Y chromosome scaffold with the rhesus macaque Y chromosome (Fig. 3). We observed a substantial amount of synteny between the putative baboon Y and the rhesus Y, comparable to what is observed between the chimpanzee Y and the human Y chromosomes. This suggests that the Panubis1.0 chromosome Y captures at least part of the true chromosome Y. (For comparison, genetic divergence between baboon and rhesus is similar to human–chimpanzee divergence [41].) The observed breaks in synteny are consistent with the well-documented high rate of chromosomal rearrangements on mammalian Y chromosomes [42].

### Genome annotation

Annotation of the protein and non-protein coding genes was performed by NCBI's RefSeq database (RefSeq, RRID:SCR_003496), based on RNA sequencing (RNA-seq) of 4 captive baboons at the SNPRC (BioProject PRJNA559725), as well as other publicly available baboon expression data. Panubis1.0 contains 21,087 protein-coding genes and 11,295 non-coding genes. This is a slight decrease in the number of protein-coding genes relative to Panu_3.0 (21,087 vs 21,300), which can be explained by merging genes together (n = 252) and an increase in the number of non-coding genes (11,295 vs 8,433). Panubis1.0 also contains slightly more pseudogenes (6,680 vs 5,998) and genes with splice variants (14,526 vs 13,693). Many of these differences may reflect insights gained from an improved assembly leading to an increased ability to map sequencing data; indeed, during genome annotation, 88% of RNA-seq reads mapped to Panubis1.0 while only 80% mapped to Panu3.0.

Overall, most genes (66%) are highly similar or identical between Panubis1.0 and Panu_3.0. Of the remaining genes, 13% of genes contain major changes (e.g., were split, moved, changed gene type, or changed substantially in completeness), 20% are novel in Panubis1.0, and 12% deprecated from Panu_3.0.

### Comparisons with the publicly available Panu_3.0 assembly

**Figure 4:**
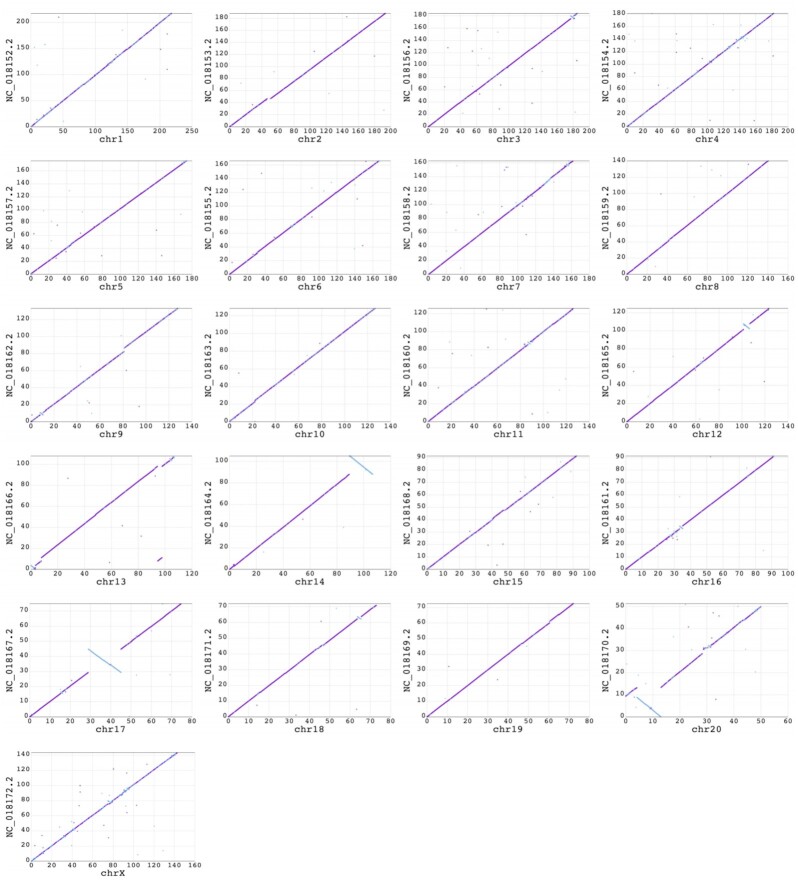
Dot plots showing alignment of Panu_3.0 reference-assisted chromosomes vs Panubis1.0 chromosome-length scaffolds. The Panu_3.0 assembly is shown on the Y-axis and the Panubis1.0 assembly is shown on the X-axis. Each dot represents the position of a syntenic block between the 2 assemblies as determined by the nucmer alignment. The color of the dot reflects the orientation of the individual alignments (purple indicates consistent orientation and blue indicates inconsistent orientation). The dot plots illustrate that there are chromosomes containing large inversions and translocations in the Panu_3.0 assembly with respect to the Panubis1.0 assembly.

**Figure 5:**
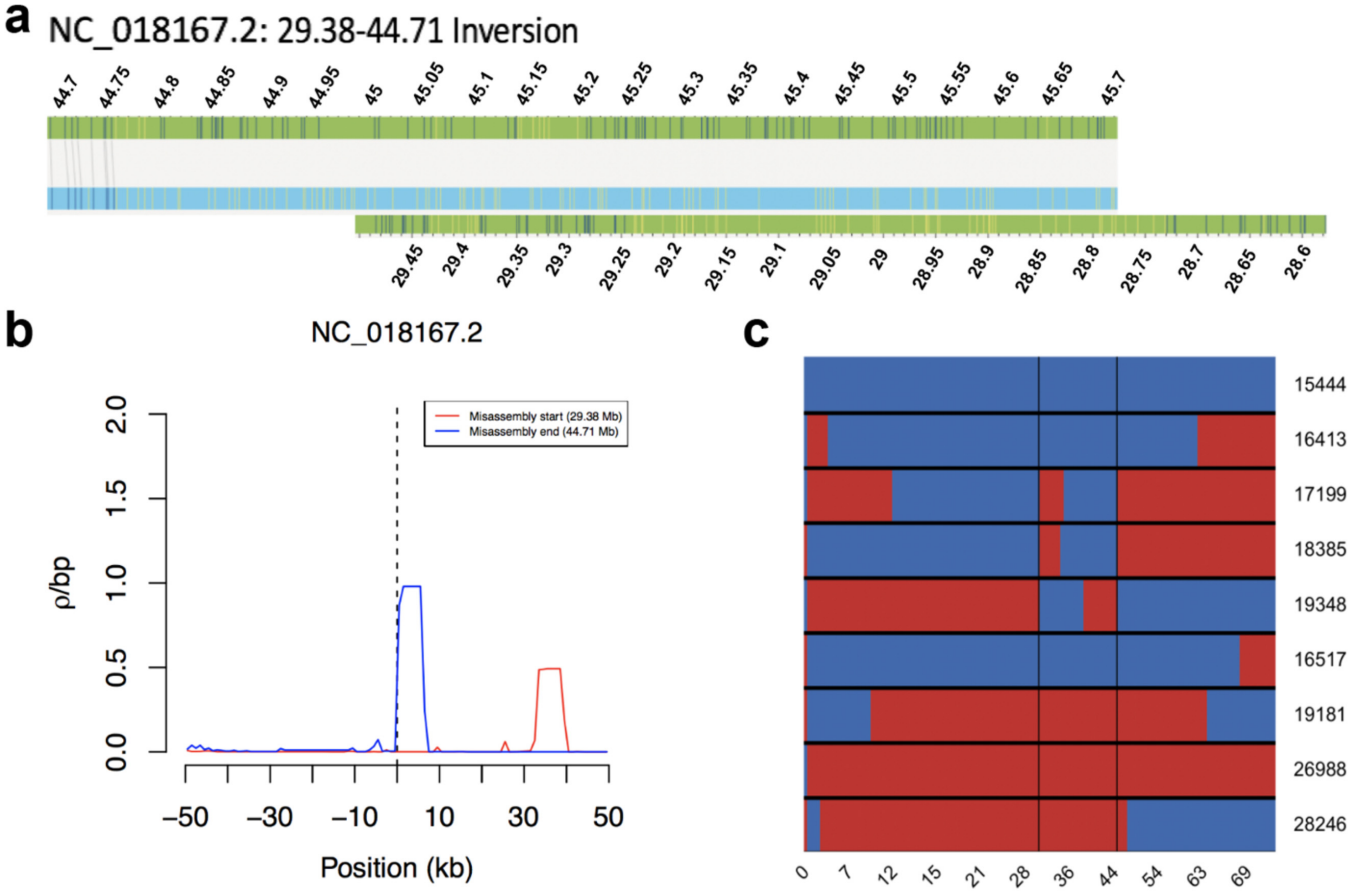
Evidence for misassembly on chromosome NC_018167.2 in Panu_3.0. (**a**) Bionano optical map alignment to the Panu_3.0 assembly demonstrates an inversion on chromosome NC_018167.2 beginning at ∼29.38 Mb and ending at ∼44.71 Mb. (**b**) Estimates of the population recombination rate ρ near the potential synteny breaks of the inversion identified on chromosome NC_018167.2. (**c**) The x-axis shows positions along chromosome NC_018167.2 in Panu_3.0, where each row represents 1 of the 9 offspring of sire 10,173. Switches between red and blue within a row represent a recombination event. The 2 vertical black lines represent locations where ≥3 recombinations occur at the same locus, indicating a potential misassembly.

Fig. 4 presents a dot plot between the chromosomes of the Panubis1.0 and the Panu_3.0 assemblies. There are chromosomes with large differences between the 2 assemblies, and these differences are evident even in the chromosome-scale dot plots. Table 2 presents a list of large (>100 kb) differences between the Panubis1.0 and Panu_3.0 assemblies where we have evidence based on Hi-C data that suggested that the Panubis1.0 assembly is correct. We used several orthogonal sources of information to assess whether these were errors in our Panubis1.0 assembly or in the Panu_3.0 assembly. These included Bionano Genomics optical maps obtained from the same individual used for generating Panubis1.0, linkage information from a pedigree of baboons that were all sequenced to high coverage, and linkage disequilibrium information from 24 unrelated olive baboons from the SNPRC pedigreed baboon colony. We manually examined each of these breaks in synteny between Panubis1.0 and Panu_3.0 to determine whether these independent sources of evidence supported one assembly over the other (summarized in Table 2). Overall, in 11 of 12 large syntenic differences between Panubis1.0 and Panu_3.0 where the Hi-C data support the Panubis1.0 assembly, ≥1 of these independent sources provided additional evidence that the Panubis1.0 assembly is correct (Fig. 5, Supplementary Figs S1–S5).

**Table 2:** Likely large (>100 kb) assembly errors in Panu_3.0, ordered by size

Panu_3.0 chromosome	Panu_3.0 (Mb)		Panu_2.0 (Mb)		Type	Linkage support	BNG support	LDhelmet support
	Start	End	Start	End				
NC_018164.2	88.05	104.99	87.61	104.98	Inv	Start^1^	Yes	Unknown^1^
NC_018167.2	29.38	44.71	29.25	44.53	Inv	Start + end	Yes	Start + end
NC_018156.2	4.04	8.67	4.18	8.63	Inv	No	Yes^2^	No
NC_018162.2	82.42	86.47	81.91	84.01	Trans	Start + end	No^3^	No
NC_018166.2	104.28	108.05	103.66	107.44	Inv	No	Yes	No
NC_018165.2	15.93	19.48	15.85	19.40	Inv	No	No	No
NC_018166.2	96.94	100.12	96.39	99.54	Trans	Start + end	Yes^4^	Start + end
NC_018160.2	36.05	36.75	35.88	36.55	Trans	No	Yes^4^	Start
NC_018163.2	23.19	23.66	0	0.47	Trans	No	Yes^2^	No
NC_018164.2	4.05	4.49	3.99	4.45	Trans	No^5^	Yes	No
NC_018165.2	100.91	101.18	100.31	100.59	Trans	No	Yes	No
NC_018152.2	166.73	166.89	169.86	170.10	Trans	Start + end	Yes	End

Note that a “no” in the “Linkage support” or “LDhelmet support” columns is inconclusive and should not be interpreted as support for the Panu_3.0 assembly being correct.

1 Unable to determine whether linkage and LDhelmet provide support at the end breakpoint due to a lack of synteny between Panu_2.0 and Panu_3.0.

2 Panu_2.0 assembly seems to be correct.

3 Bionano Genomics (BNG) maps do not support a translocation with these breakpoints. However, they do support a potential large structural variant at the starting breakpoint.

4 BNG maps support the presence of a large structural variant, which may be a translocation.

5 Linkage data suggest a potential polymorphic inversion (in 16,413) partially overlapping with this interval.

Table 3 presents an additional list of large inversion differences between Panubis1.0 and Panu_3.0, where, on the basis of the current data, it is difficult to conclude whether it is Panubis1.0 or Panu_3.0 that is correct. For these regions, Hi-C data only weakly support the Panubis1.0 assembly and do not provide direct evidence that the Panu_3.0 assembly is incorrect. In addition, the aforementioned orthogonal sources of information are inconclusive as to which assembly is correct for each of these regions. Further research will be needed to assess the correct orientation of the baboon genome sequence in each of these problematic regions.

**Table 3:** Additional large (>100 kb) inversion differences between Panubis1.0 and Panu_3.0, ordered by size

Panubis1.0 chromosome	Panubis1.0 Start (Mb)	Panubis1.0 End (Mb)	Panu_3.0 chromosome	Panu_3.0 chromosome	Panu_3.0 Start (Mb)	Panu_3.0 End (Mb)
NC_044992.1	28.89	45.01	CM001506.2	NC_018167.2	29.38	44.79
NC_044995.1	0.00	13.00	CM001509.2	NC_018170.2	0.00	13.31
NC_044987.1	101.26	106.48	CM001504.2	NC_018165.2	101.44	107.53
NC_044978.1	176.83	181.37	CM001495.2	NC_018156.2	175.08	180.09
NC_044986.1	86.61	90.73	CM001499.2	NC_018160.2	85.56	90.30
NC_044988.1	0.00	3.50	CM001505.2	NC_018166.2	0.00	3.78
NC_044996.1	86.67	89.58	CM001511.2	NC_018172.2	86.91	90.23
NC_044982.1	154.35	156.82	CM001497.2	NC_018158.2	155.71	158.53
NC_044984.1	7.96	10.58	CM001501.2	NC_018162.2	8.03	10.83
NC_044991.1	33.09	35.09	CM001500.2	NC_018161.2	32.46	35.05
NC_044996.1	93.67	95.52	CM001511.2	NC_018172.2	94.22	96.59
NC_044981.1	68.61	71.05	CM001494.2	NC_018155.2	69.37	71.65
NC_044996.1	40.49	42.78	CM001511.2	NC_018172.2	41.15	43.34
NC_044996.1	10.01	11.79	CM001511.2	NC_018172.2	10.20	12.06
NC_044996.1	31.80	33.37	CM001511.2	NC_018172.2	32.11	33.97
NC_044979.1	142.32	144.05	CM001493.2	NC_018154.2	141.96	143.71
NC_044996.1	90.77	92.54	CM001511.2	NC_018172.2	91.42	92.99
NC_044993.1	63.59	65.52	CM001510.2	NC_018171.2	62.31	63.73
NC_044991.1	26.79	28.49	CM001500.2	NC_018161.2	26.52	27.82
NC_044980.1	0.02	0.78	CM001496.2	NC_018157.2	0.02	1.26
NC_044979.1	0.00	0.73	CM001493.2	NC_018154.2	0.00	0.75

We cannot definitively determine which orientation is correct for these inversions, and they should be considered provisional.

### Linkage disequilibrium analyses

We estimated the scaled recombination rate ρ (=4*Nr*, where *N* is the effective population size and *r* is the recombination rate per generation) using LDhelmet [43] from 24 unrelated olive baboons [44]. We then identified potential breaks in synteny as regions with total ρ > 500 and ρ/bp > 0.2. We considered there to be evidence of a synteny break if 1 of these regions was within 50 kb of a potential breakpoint (as identified in Panu_3.0 vs. Panubis1.0 comparisons). The false discovery rate for this definition is ∼4%.

To calculate recombination rates, we used a variant call set mapped onto the old assembly Panu_2.0, as described in Robinson et al. [44]. For the potential breaks in synteny identified above, we used liftover to convert the breakpoints into Panu_3.0 coordinates and verified that Panu_2.0 and Panu_3.0 were syntenic with each other across the breakpoints.

Finally, owing to the inherent noise in linkage disequilibrium–based estimates of ρ, the lack of evidence for a synteny break in Panu_3.0 is not positive evidence that the Panu_3.0 assembly is correct.

### Inference of crossovers in a baboon pedigree

**Figure 6:**
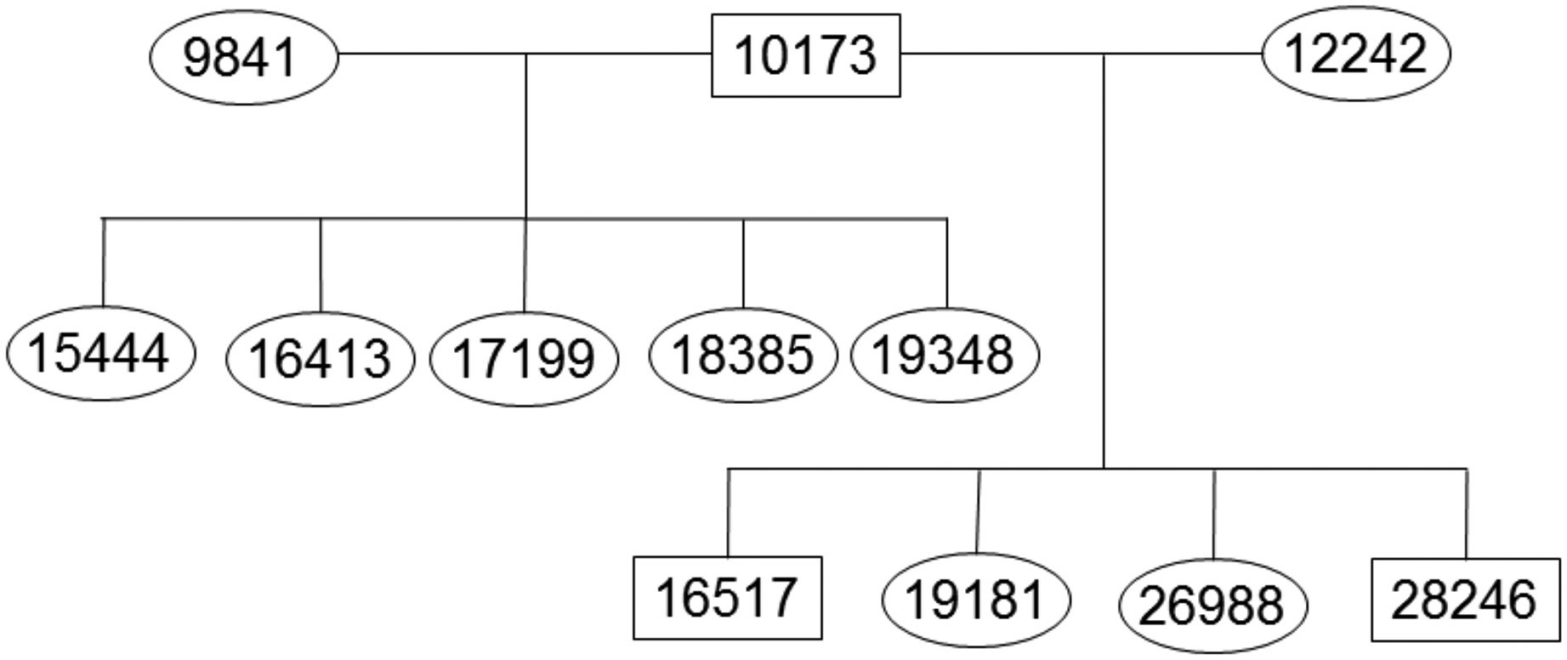
Pedigree of baboons used in linkage analysis. Circles represent females, and squares, males.

We used a previously described vcf file for the baboons shown in Fig. 6, which was mapped using Panu_2.0 coordinates and lifted over to Panu_3.0 coordinates. We considered only biallelic single-nucleotide polymorphisms (SNPs), and required a depth ≥15, QUAL >50, and genotype quality ≥40 to make a genotype call. We further required an allelic balance of >0.3 for heterozygote calls and <0.07 for homozygote calls, and excluded all repetitive regions as described in [44].

We focused our analyses on those SNPs that were most informative about recent crossover events. For example, to detect paternal crossovers, we restricted our analyses to SNPs where 10,173 was heterozygous, both 9,841 and 12,242 were homozygous, and all 9 offspring had genotype calls. (For maternal crossovers, we required 10,173 to be homozygous and both 9,841 and 12,242 to be heterozygous.) For these sites, it is straightforward to infer which allele (coded as 0 for reference allele and 1 for alternative allele) was passed on from 10,173 to his offspring. While the haplotypic phase of 10,173 is unknown, we can infer crossover events on the basis of the minimum number of crossovers needed to be consistent with the observed patterns of inheritance in the offspring of 10,173 [45]. For example, Fig. 5c shows that the inheritance pattern near position 29.38 requires ≥3 crossovers (e.g., in individuals 17,199, 18,385, and 19,348).

For each potential error in the Panu_3.0 assembly, we converted the breakpoint location into Panu_2.0 coordinates and verified synteny between Panu_2.0 and Panu_3.0 across the breakpoint region. We then determined whether there were an abnormally large number of crossovers inferred right at the breakpoint. Specifically, if we inferred ≥3 crossover events (out of 18 total meioses, 9 paternal and 9 maternal), then we considered this as evidence that the Panu_3.0 assembly is incorrect, as in Fig. 5c (cf. “Linkage upport” column in Table 2). Note that the converse is not true: <3 inferred crossover events is not evidence that the Panu_3.0 assembly is correct at a particular location.

### Repeat analysis

We analyzed the repeat content of the Panubis1.0 and Panu_3.0 genome assemblies using RepeatMasker (RepeatMasker, RRID:SCR_012954) [46] version open-4.0.8 in sensitive mode and with blastp version 2.0MP-WashU using the RepeatMasker Combined Database: Dfam_Consensus-20181026, RepBase-20181026. The following parameters were used to run RepeatMasker: RepeatMasker -engine wublast -species “papio anubis” -s -no_is -cutoff 255 -frag 20 000.

Fig. S6 summarizes the distribution of various types of repeats found in the 2 genome assemblies. We found that the genome assemblies are comparable in terms of their repeat content.

## Conclusion

The development and commercialization of new technologies by companies such as Illumina, 10x Genomics, Bionano Genomics, Dovetail Genomics, and Phase Genomics has enabled researchers to cheaply generate fully *de novo* genome assemblies with high scaffold contiguity (e.g., [36, 39, 47–49]). When used in combination with long-read sequences (e.g., from Oxford Nanopore or Pacific Biosciences), these technologies can produce high-quality genome assemblies at a fraction of the cost of traditional clone library–based approaches (e.g., [48, 50]). In this context, our assembly Panubis1.0 provides a 10-fold increase in contig N50 size and a 240-fold increase in scaffold N50 size relative to Panu_3.0 at <1% of the reagent cost. The contiguity of this assembly will be especially useful for future studies where knowing the genomic location is important (e.g., hybridization or recombination studies).

One natural question that arises with any new genome assembly is how one assesses that an assembly is *“*correct*.”* Indeed, some of the recently published Hi-C–based assemblies have not provided any corroborating evidence supporting their assemblies (e.g., [51]). Here, we used 3 independent sources of information to provide evidence that 11 of 12 large syntenic differences identified from the dot plots are correct in our new baboon assembly (Panubis1.0) relative to the previous assembly Panu_3.0 (Table 2). In all, the incorporation of optical maps and linkage and linkage disequilibrium data provide substantially more support for our assembly than was produced by previous Hi-C–based assemblies (e.g., [48–50]) and counters any potential criticism of the fact that our genome assembly (using individual “15944” from the SNPRC baboon colony) comes from a different individual from the previous baboon assembly (individual 1 × 1155 from the SNPRC baboon colony).

There is however a larger list of 21 inversion differences between Panubis1.0 and Panu_3.0 where the Hi-C data do not provide definitive evidence on which orientation is correct (Table 3). While Hi-C–based assemblies may be prone to small contig inversions within scaffolds, this should be less of a problem for the large inversions outlined here because there will be few interactions that span the full length of the contig, and the correct orientation is generally apparent from the higher weight of links. These changes to the baboon assembly should be considered provisional until additional data can be collected (e.g., high-coverage long-read data) that provide a more definitive answer.

## Data Availability

All of the raw sequence data from individual 15,944, as well as the Panubis1.0 assembly, are available without restriction from NCBI under BioProject PRJNA527874. New RNA-seq data used for genome annotation are available under BioProject PRJNA559725. The genome annotation report and raw files can be found at [52]. All supporting data and materials are available in the *GigaScience* GigaDB database [53].

## Additional Files

**Supplemental Figure S1:**Linkagedisequilibrium–based evidence for misassemblies in Panu_3.0. Estimates of the population recombination rate ρ near the potential synteny breaks of the misassemblies identified in chromosomes (a) NC_018166.2, (b) NC_018160.2, and (c) NC_018152.2. Red represents the beginning of a misassembly event, and blue, the end of a misassembly event.

**Supplemental Figure S2:**Recombination-based evidence for misassemblies in Panu_3.0. Shown on the x-axis are positions along chromosomes in Panu_3.0, where each row represents 1 of the 9 offsprings of sire 10173. Switches between red and blue within a row represent a recombination event. The vertical black lines represent locations where ≥3 recombinations occur at the same locus, indicating a potential misassembly; except in (d), where recombination occurs at ∼167 Mb but is not shown by a vertical black line.

**Supplemental Figure S3:**Evidence for inversions in Panu_3.0 based on Bionano alignment. (a) Inversion on chromosome NC_018164.2 demonstrated by Bionano optical map alignment. (b) Alignment to Bionano optical map shows inverted coordinates due to an inversion on chromosome NC_018156.2. (c) Bionano optical map alignment shows an inversion on chromosome NC_018166.2.

**Supplemental Figure S4:**Evidence for translocations in Panu_3.0 based on Bionano alignment. (a) Breaks in Bionano alignment on chromosome NC_018166.2 indicate a misassembly. (b) Bionano optical map alignment demonstrates a misassembly on chromosome NC_018160.2. (c) Bionano optical map alignment shows a translocation between chromosomes NC_018163.2 and NC_018164.2.

**Supplemental Figure S5:**Evidence for translocations in Panu_3.0 based on Bionano alignment. (a) Breaks in Bionano alignment on chromosome NC_018164.2 indicate a misassembly. (b) Bionano optical map alignment demonstrates a misassembly on chromosome NC_018165.2. (c) Bionano optical map alignment shows a translocation on chromosome NC_018152.2.

giaa134_GIGA-D-20-00013_Original_Submission

giaa134_GIGA-D-20-00013_Revision_1

giaa134_GIGA-D-20-00013_Revision_2

giaa134_GIGA-D-20-00013_Revision_3

giaa134_Response_to_Reviewer_Comments_Original_Submission

giaa134_Response_to_Reviewer_Comments_Revision_1

giaa134_Response_to_Reviewer_Comments_Revision_2

giaa134_Reviewer_1_Report_Original_Submission Xiao-Guang Qi -- 3/15/2020 Reviewed

giaa134_Reviewer_1_Report_Revision_1 Xiao-Guang Qi -- 8/15/2020 Reviewed

giaa134_Reviewer_2_Report_Original_Submission Taras K Oleksyk, Ph.D. -- 4/10/2020 Reviewed

giaa134_Reviewer_2_Report_Revision_1 Taras K Oleksyk, Ph.D. -- 9/1/2020 Reviewed

giaa134_Supplemental_File

## Abbreviations

BLAST: Basic Local Alignment Search Tool; bp: base pairs; BUSCO: Benchmarking Universal Single-Copy Orthologs; Gb: gigabase pairs; kb: kilobase pairs; Mb: megabase pairs; NCBI: National Center for Biotechnology Information; NIH: National Institutes of Health; RNA-seq: RNA sequencing; SNP: single-nucleotide polymorphism; SNPRC: Southwest National Primate Research Center.

## Competing Interests

The authors declare that they have no competing interests.

## Funding

The work was supported in part by NIH grants R24 OD017859 (to L.A.C. and J.D.W.), R01 GM115433 (to J.D.W.), R01 GM094402 (to Y.S.S.), R35 GM134922 (to Y.S.S.), R01 HG005946 (to P.Y.K.) and by a Packard Fellowship for Science and Engineering (to Y.S.S.). Y.S.S. is a Chan Zuckerberg Biohub Investigator.

## Authors' Contributions

J.D.W., L.A.C., and Y.S.S. conceived the project. J.G., S.D., S.S., M.L.S., and P.Y.K. generated data for the project. M.L.S. and S.S.B. performed the genome assembly. S.S.B., M.L.S., J.R., T.P.V., and J.D.W. performed the other analyses. S.S.B. and J.D.W. wrote the manuscript with contributions from all authors.
