# Supplementary material for: Accurate assembly of the olive baboon (Papio anubis) genome using long-read and Hi-C data
Source: Gigascience. 2020 Dec 7;9(12):giaa134. doi: 10.1093/gigascience/giaa134 (PMC7719865; doi:10.1093/gigascience/giaa134)
Supplement: giaa134_GIGA-D-20-00013_Revision_2 [file giaa134_giga-d-20-00013_revision_2.pdf]

GigaScience

Accurate assembly of the olive baboon (Papio anubis) genome using long-read and Hi-C data

--Manuscript Draft--

**Manuscript Number:**	GIGA-D-20-00013R2
**Full Title:**	Accurate assembly of the olive baboon (Papio anubis) genome using long-read and Hi-C data
**Article Type:**	Data Note
**Funding Information:**	National Institutes of Health (US) (R24 OD017859) — Dr. Laura A. Cox, Dr. Jeffrey D. Wall<br>National Institutes of Health (US) (R01 GM094402) — Dr. Yun S. Song<br>National Institutes of Health (US) (R01 HG005946) — Dr. Pui-Yan Kwok<br>David and Lucile Packard Foundation (Packard Fellowship for Science and Engineering) — Dr. Yun S. Song<br>National Institutes of Health (R01 GM115433) — Dr. Jeffrey D. Wall<br>National Institutes of Health (R35 GM134922) — Dr. Yun S. Song
**Abstract:**	Background<br><br>Baboons are a widely used nonhuman primate model for biomedical, evolutionary and basic genetics research. Despite this importance, the genomic resources for baboons are quite limited. In particular, the current baboon reference genome Panu_3.0 is a highly fragmented, reference-guided (i.e., not fully de novo) assembly, and its poor quality inhibits our ability to conduct downstream genomic analyses.<br><br>Findings<br><br>Here we present a truly de novo genome assembly of the olive baboon (Papio anubis) that uses data from several recently developed single-molecule technologies. Our assembly, Panubis1.0, has an N50 contig size of ~1.46 Mb (as opposed to 139 Kb for Panu_3.0), has single scaffolds that span each of the 20 autosomes and the X chromosome, and is freely available for scientific use from NCBI.<br><br>Conclusions<br><br>We present multiple lines of evidence (including Bionano Genomics data, linkage information, and patterns of linkage disequilibrium) suggesting that the Panubis1.0 assembly corrects large assembly errors in Panu_3.0. This in turn has led to an improved baboon annotation, making Panubis1.0 much more useful for future genomic studies.
**Corresponding Author:**	Jeffrey D. Wall<br><br>UNITED STATES
**Corresponding Author Secondary Information:**	
**Corresponding Author's Institution:**	
**Corresponding Author's Secondary Institution:**	
**First Author:**	Sanjit Singh Batra
**First Author Secondary Information:**	

Powered by Editorial Manager® and ProduXion Manager® from Aries Systems Corporation

**Order of Authors:**	Sanjit Singh Batra
	Michal Levy-Sakin
	Jacqueline Robinson
	Joseph Guillory
	Steffen Durinck
	Tauras P. Vilgalys
	Pui-Yan Kwok
	Laura A. Cox
	Somasekar Seshagiri
	Yun S. Song
	Jeffrey D. Wall
**Order of Authors Secondary Information:**	
**Response to Reviewers:**	We thank the reviewer for their comments. We provide our response to their suggestions below.<br><br>Reviewer #1:<br><br>- The description of the repeats is still very simple. Despite that total length of repeats by RepeatMasker are present in supplementary figure, this is not enough. Much details are missing, do they perform this based on homology or de novo? maybe both. From their results, the repeats for Panubis1.0 is not higher even lower that Panu_3.0. This is strange, please make some new efforts to provide more evidences. I do not know if the de novo method will work but the authors should try it.<br><br>The repeat analysis was done by running RepeatMasker version open-4.0.8 in sensitive mode and was run with blastp version 2.0MP-WashU using the RepeatMasker Combined Database: Dfam_Consensus-20181026, RepBase-20181026. The following parameters were used to run RepeatMasker: RepeatMasker -engine wublast -species 'papio anubis' -s -no_is -cutoff 255 -frag 20000<br><br>We have also included these details into the manuscript.<br><br><br>- The authors used the Euarchontoglires gene set instead of the broader Mammalia gene set provided by BUSCO. However, the result is not very good. At least, no new evidence to show the high quality of their genome. I do not know if this are related with their assembly strategy. I am wondering if the 15x ONT coverage could be used for assembly directly, it is hard to estimate from the sequence coverage. But the authors should think about this carefully and some more work needed to be done to fix this problem.<br><br>The Canu assembler documentation (which can be found at https://canu.readthedocs.io/en/latest/quick-start.html) recommends that "For eukaryotic genomes, coverage more than 20x is enough to outperform current hybrid methods, however, between 30x and 60x coverage is the recommended minimum." Since we only had 15x nanopore reads, we consequently didn't attempt to assemble the nanopore reads de novo and opted to use them for scaffolding of contigs instead. This reasoning is outlined in the discussion section.
**Additional Information:**	
**Question**	**Response**
Are you submitting this manuscript to a special series or article collection?	No
**Experimental design and statistics**	Yes

Powered by Editorial Manager® and ProduXion Manager® from Aries Systems Corporation

Full details of the experimental design and statistical methods used should be given in the Methods section, as detailed in our Minimum Standards Reporting Checklist. Information essential to interpreting the data presented should be made available in the figure legends.<br><br>Have you included all the information requested in your manuscript?	
**Resources**<br><br>A description of all resources used, including antibodies, cell lines, animals and software tools, with enough information to allow them to be uniquely identified, should be included in the Methods section. Authors are strongly encouraged to cite Research Resource Identifiers (RRIDs) for antibodies, model organisms and tools, where possible.<br><br>Have you included the information requested as detailed in our Minimum Standards Reporting Checklist?	Yes
**Availability of data and materials**<br><br>All datasets and code on which the conclusions of the paper rely must be either included in your submission or deposited in publicly available repositories (where available and ethically appropriate), referencing such data using a unique identifier in the references and in the "Availability of Data and Materials" section of your manuscript.<br><br>Have you have met the above requirement as detailed in our Minimum Standards Reporting Checklist?	Yes

Manuscript

Click here to access/download;Manuscript;FINAL_Manuscript_without_figures

Accurate assembly of the olive baboon (*Papio anubis*) genome using long-read and Hi-C data

Sanjit Singh Batra[1], Michal Levy-Sakin[2], Jacqueline Robinson[3], Joseph Guillory[4], Steffen Durinck[4,5], Tauras P. Vilgalys[6], Pui-Yan Kwok[2,3], Laura A. Cox[7,8], Somasekar Seshagiri[4], Yun S. Song[1,9,10] and Jeffrey D. Wall[3,*]

[1]Computer Science Division, University of California, Berkeley, CA 94720;
[2]Cardiovascular Research Institute, University of California San Francisco, San Francisco, CA 94143;
[3]Institute for Human Genetics, University of California San Francisco, San Francisco, CA 94143;
[4]Department of Molecular Biology, Genentech, Inc., 1 DNA Way, South San Francisco, CA 94080;
[5]Bioinformatics and Computational Biology Department, Genentech, Inc., 1 DNA Way, South San Francisco, CA 94080;
[6]Department of Evolutionary Anthropology, Duke University, Durham, NC 27705
[7]Center for Precision Medicine, Department of Internal Medicine, Section of Molecular Medicine, Wake Forest School of Medicine, Winston-Salem, NC 27101
[8]Southwest National Primate Research Center, Texas Biomedical Research Institute, San Antonio, TX 78245
[9]Department of Statistics, University of California, Berkeley, CA 94720;
[10]Chan Zuckerberg Biohub, San Francisco, CA 94158

*Corresponding Author: Jeff.Wall@ucsf.edu

ABSTRACT

Background

Baboons are a widely used nonhuman primate model for biomedical, evolutionary and basic genetics research. Despite this importance, the genomic resources for baboons are quite limited. In particular, the current baboon reference genome Panu_3.0 is a highly fragmented, reference-guided (i.e., not fully *de novo*) assembly, and its poor quality inhibits our ability to conduct downstream genomic analyses.

Findings

Here we present a truly *de novo* genome assembly of the olive baboon (*Papio anubis*) that uses data from several recently developed single-molecule technologies. Our assembly, Panubis1.0, has an N50 contig size of ~1.46 Mb (as opposed to 139 Kb for Panu_3.0), and has single scaffolds that span each of the 20 autosomes and the X chromosome.

Conclusions

We highlight multiple lines of evidence (including Bionano Genomics data, pedigree linkage information, and linkage disequilibrium data) suggesting that there are several large assembly errors in Panu_3.0, which have been corrected in Panubis1.0.

Data Description

Introduction

Baboons are ground-living monkeys native to Africa and the Arabian Peninsula. Due to their relatively large size, abundance and omnivorous diet, baboons have increasingly become a major biomedical model system (reviewed in [1]). Baboon research has been facilitated by the creation (in 1960) and maintenance of a large, pedigreed, well-phenotyped baboon colony at the Southwest National Primate Research Center (SNPRC) and an ability to control the environment of subjects in ways that are obviously not possible in human biomedical studies. For example, baboons have been used to study the effect of diet on cholesterol and triglyceride levels in controlled experiments where all food consumption is completely controlled [2] [3] [4]. In recent years, linkage studies in baboons have helped identify genetic regions affecting a wide range of phenotypes, such as cholesterol levels [5] [6], estrogen levels [7], craniofacial measurements [8], bone density [9] [10] and lipoprotein metabolism [11]. In addition, studies have also documented that the genetic architecture of complex traits in baboons can be directly informative about analogous traits in humans (e.g.,[10] [12]). In parallel, baboons have been widely used in studies of animal behavior and evolution. For example, the Amboseli Baboon Research Project has studied wild baboon troops continuously since 1971, and produced roughly 300 scientific publications (cf. https://amboselibaboons.nd.edu), including the first study of whole-genome sequence data in baboons [26].

The success of these and other studies have been mediated in part by recent advances in molecular genetics technologies. In particular, the ability to cheaply genotype and/or sequence samples of interest has led to a revolution in genetic studies of the associations between genotype and phenotype. While human genetic studies now routinely include the analyses of whole-genome sequence data from many thousands of samples (e.g., [13] [14] [15] [16] [17]), comparable studies in model organisms have lagged far behind. Part of the reason for this is the lack of genetic resources in non-human species. Large, international projects such as the Human Genome Project [18] [19], International HapMap Project [20] [21] [22] and the 1000 Genomes Project [23] [24] [25] have provided baseline information on sequences and genetic variation, and subsequent human genetic studies have utilized this background information.

The first published baboon genome assembly was from a yellow baboon [26]. This assembly used a combination of Illumina paired-end and Illumina mate-pair sequence data (with mean library insert sizes ranging from 175 bp to 14 Kbp) to produce a highly fragmented assembly with contig N50 of 29 Kbp and scaffold N50 of 887 Kbp. The public olive baboon assembly, Panu_3.0, suffers from the same problem of having small contigs and scaffolds (contig N50 of 139 Kbp and *de novo* scaffold N50 of 586 Kbp) [27]. The authors of the public olive baboon assembly chose to distribute a reference-guided assembly with scaffolds mapped onto rhesus (*Macaca mulatta*) chromosomes. As a consequence, any syntenic differences between rhesus and baboon will result in large-scale assembly errors in Panu_3.0. One additional drawback of this baboon

genome assembly was its informal embargo from 2008 to 2019 under the guidelines of the Fort Lauderdale agreement. Hence, its influence on scientific research has been negligible.

In this project, we focus on providing a high-quality, *de novo* genome assembly for olive baboon (*Papio anubis*), which we call Panubis1.0, with the hope that this resource will enable future high-resolution genotype-phenotype studies. Unlike previous baboon genome assembly efforts, we use a combination of three recently developed technologies (from 10x Genomics linked-reads, Oxford Nanopore long reads, and Hi-C) to increase the long-range contiguity of our assembly. These newly developed technologies enable us to generate assemblies where the autosomes (and the X chromosome) are each spanned by a single scaffold at a cost that is orders of magnitude cheaper than the Panu_3.0 assembly. We also verify that many of the large-scale syntenic differences between our Panubis1.0 and Panu_3.0 are due to errors in the public assembly rather than our own. Our assembly is available for scientific use without any restrictions.

Genome Sequencing

Index animal: We used individual number 15944 (currently deceased) from the SNPRC pedigreed baboon colony for all of the sequencing and genome assembly work associated with this project.

10x Genomics sequencing: High molecular weight genomic DNA extraction, sample indexing, and generation of partition barcoded libraries were performed according to the 10x Genomics (Pleasanton, CA, USA) Chromium Genome User Guide and as published previously ([28]). An average depth of ~60X was produced and analyzed for this project.

Oxford Nanopore sequencing: Libraries for the Oxford Nanopore sequencing were constructed as described previously ([45]) using DNA derived from whole blood. The sequencing was conducted at Genentech, Inc. (South San Francisco, CA, USA); we analyzed data with an average depth of ~15X for this project.

Bionano optical maps: High-molecular-weight DNA was extracted, nicked, and labeled using the enzyme Nt.BspQI (New England Biolabs (NEB), Ipswich, MA, USA), and imaged using the Bionano Genomics Irys system (San Diego, CA, USA) to generate single-molecule maps for assessing breaks in synteny between Panu_3.0 and Panubis1.0.

Hi-C sequencing: High molecular weight DNA from Jenny Tung (Duke University) was sent to Phase Genomics. ~15X Hi-C data was obtained using previously described techniques [46].

Genome Assembly

The main strength of our approach is in combining data from multiple platforms (10x Genomics linked-reads, Oxford Nanopore long-reads, Illumina paired-end short-reads, and Hi-C), which have complementary advantages. Figure 1 describes our assembly strategy. We began by assembling 10x Genomics reads generated with their Chromium system (average depth ~60x) using the SUPERNOVA assembler (version 1.1, default parameters) [28], which yielded an assembly with a contig N50 of ~84 kb and a scaffold N50 of ~15.7 Mb (Table 1). The gap lengths between the contigs in a scaffold obtained by assembling 10x linked-reads are arbitrary [29]. Hence, in order to leverage the Oxford Nanopore long-reads for gap-closing, we split the 10X scaffolds at every stretch of non-zero N's to obtain a collection of contigs.

We scaffolded the resulting contigs with Oxford Nanopore long-reads (average depth ~15X) using the LR_Scaf (version 1.1.4, default parameters) [30] scaffolding method. (In accordance with the Canu assembler documentation, available at https://canu.readthedocs.io/en/latest/quick-start.html, we did not have a sufficient depth of coverage to perform *de novo* assembly directly from the Nanopore reads.) This resulted in an assembly with a contig N50 of ~134 kb and a scaffold N50 of ~1.69 Mb (Table 1). These resulting scaffolds are more amenable to gap-closing, because the gap lengths (number of Ns between two consecutive contigs) are estimated by long-reads that span each gap and align to the flanking regions of that gap.

Upon performing gap-closing with the same set of Oxford Nanopore long-reads using LR_Gapcloser (v1.1, default parameters) [31], we obtained an assembly with a contig N50 of ~1.47 Mb and a scaffold N50 of ~1.69 Mb (Table 1). Note that this increase in contig N50 of ~84Kb from the 10x Genomics linked-read assembly, to a contig N50 of ~1.47 Mb, would not have been possible if we had simply performed gap-closing with the Oxford Nanopore long reads directly on the 10x-based assembly without first splitting it into its constituent contigs. Finally, we polished the resulting assembly by aligning Illumina paired-end reads (average depth ~60X in PE150 reads) using Pilon (version 1.22, default parameters) [32].

In order to scaffold the resulting assembly with Hi-C data, we first set aside scaffolds shorter than 50 kb, which comprised only ~1.8% of the total sequence base pairs. This was done because Hi-C based scaffolding is more reliable for longer scaffolds, since there are more Hi-C reads aligning to longer scaffolds. We then ordered and oriented the remaining scaffolds using the 3D *de novo* assembly (3d-dna) pipeline (version 180419, default parameters) [33] using ~15X Hi-C data generated by Phase Genomics [34]. Finally, we manually corrected misassemblies in the resulting Hi-C based assembly by visualizing the Hi-C reads aligned to the assembly, using Juicebox Assembly Tools (version 1.6.11) [35], following the strategy described in [36]. Figure 2 shows Hi-C reads aligned to the resulting assembly with the blue squares on the diagonal representing chromosomes.

The resulting *Papio anubis* genome assembly, which we name Panubis1.0, contains ~2.87 Gb of sequenced base pairs (non-N base pairs) and 2.3 Mb (<0.1%) of gaps (N's). Single scaffolds spanning the 20 autosomes and the X chromosome together

contain 95.14% (~2.73 Gb) of the sequenced base pairs. We number the autosomes as chr1 to chr20, in decreasing order of the scaffold length, so some chromosome numbers in our convention are different from Panu_3.0's numbering. We note that Panubis1.0 has a contig N50 of 1.46 Mb, which is a greater than ten-fold improvement over the contig N50 (~139 kb) of the Panu_3.0 assembly. As a result, Panubis1.0 contains five times fewer scaffolds (11,145 scaffolds with a scaffold N50 of ~140 Mb) compared to the Panu_3.0 assembly (63,235 scaffolds with a scaffold N50 of ~586 Kb); see Table 1 for a further comparison of the two assemblies. Gene completion analysis of the assembly using BUSCO (version 3, default parameters) and the euarchontoglires odb9 ortholog dataset [37] suggests that chromosomes in the Panubis1.0 assembly contain 5167 / 6192 (83.4%) *complete* genes, comparable to 5166 / 6192 (83.4%) *complete* genes found in the chromosomes of the Panu_3.0 assembly. Further, the chromosomes in the Panubis1.0 assembly contained 247 / 6192 (4.0%) *fragmented* genes, comparable to 262 / 6192 (4.2%) *fragmented* genes in the chromosomes of the Panu_3.0 assembly.

Y chromosome assembly

The Hi-C scaffolding with 3d-dna yielded an ~8 Mb scaffold that putatively represents part of the baboon Y chromosome. Since, rhesus macaque is the phylogenetically closest species to baboons which has a chromosome-scale assembly, we aligned this putative baboon Y chromosome scaffold with the rhesus macaque Y chromosome (Figure 3). We observed a substantial amount of synteny between the putative baboon Y and the rhesus Y, comparable to what is observed between the chimpanzee Y and the human Y chromosomes. This suggests that the Panubis1.0 chromosome Y captures at least part of the true chromosome Y. (For comparison, genetic divergence between baboon and rhesus is similar to human – chimpanzee divergence [38].) The observed breaks in synteny are consistent with the well-documented high rate of chromosomal rearrangements on mammalian Y chromosomes [39].

Genome Annotation

Annotation of the protein and non-protein coding genes was performed by NCBI (Reference sequence (RefSeq) database at NCBI), based on RNA sequencing of 4 captive baboons at the SNPRC (BioProject PRJNA559725) as well as other publicly available baboon expression data. Panubis1.0 contains 21,087 protein-coding genes and 11,295 non-coding genes. This is a slight decrease in the number of protein-coding genes relative to Panu_3.0 (21,087 vs 21,300) which can be explained by merging genes together (n=252), and an increase in the number of non-coding genes (11,295 vs 8,433). Panubis1.0 also contains slightly more pseudogenes (6,680 vs 5,998) and genes with splice variants (14,526 vs 13,693). Many of these differences may reflect insights gained from an improved assembly leading to an increased ability to map sequencing data; indeed, during genome annotation, 88% of RNA-seq reads mapped to Panubis1.0 while only 80% mapped to Panu3.0.

Overall, most genes (66%) are highly similar or identical between Panubis1.0 and Panu_3.0. Of the remaining genes, 13% of genes contain major changes (e.g., were split, moved, changed gene type, or changed substantially in completeness), 20% are novel in Panubis1.0, and 12% deprecated from Panu_3.0.

Comparisons with the publicly available Panu_3.0 assembly

Figure 4 presents a dotplot between the chromosomes of the Panubis1.0 and the Panu_3.0 assemblies. There are chromosomes with large differences between the two assemblies and these differences are evident even in the chromosome-scale dotplots. Table 2 presents a list of large (>100 Kb) differences between the Panubis1.0 and Panu_3.0 assemblies where we have evidence based on Hi-C data which suggested that the Panubis1.0 assembly is correct. We used several orthogonal sources of information to assess whether these were errors in our Panubis1.0 assembly or in the Panu_3.0 assembly. These included Bionano Genomics optical maps obtained from the same individual used for generating Panubis1.0, linkage information from a pedigree of baboons that were all sequenced to high coverage, and linkage-disequilibrium information from 24 unrelated olive baboons from the SNPRC pedigreed baboon colony. We manually examined each of these breaks in synteny between Panubis1.0 and Panu_3.0 to determine whether these independent sources of evidence supported one assembly over the other (summarized in Table 2). Overall, in 11 out of 12 large syntenic differences between Panubis1.0 and Panu_3.0 where the Hi-C data supports the Panubis1.0 assembly, at least one of these independent sources provided additional evidence that the Panubis1.0 assembly is correct (Figure 5, Supplementary Figures S1-S5).

Table 3 presents an additional list of large inversion differences between Panubis1.0 and Panu_3.0 where, based on the current data, it is difficult to conclude which one of Panubis1.0 and Panu_3.0 is correct. For these regions, Hi-C data only weakly support the Panubis1.0 assembly, and do not provide direct evidence that the Panu_3.0 assembly is incorrect. In addition, the orthogonal sources of information described above are inconclusive as to which assembly is correct for each of these regions. Further research will be needed to assess the correct orientation of the baboon genome sequence in each of these problematic regions.

Linkage disequilibrium analyses

We estimated the scaled recombination rate ρ (= 4Nr where N is the effective population size and r is the recombination rate per generation) using LDhelmet [47] from 24 unrelated olive baboons [48]. We then identified potential breaks in synteny as regions with total ρ > 500 and ρ / bp > 0.2. We considered there to be evidence of a synteny break if one of these regions was within 50 Kb of a potential breakpoint (as

identified in Panu_3.0 vs. Panubis1.0 comparisons). The false discovery rate for this definition is ~4%.

To calculate recombination rates, we used a variant call set mapped onto the old assembly Panu_2.0, as described in [48]. For the potential breaks in synteny identified above, we used liftover to convert the breakpoints into Panu_3.0 coordinates and verified that Panu_2.0 and Panu_3.0 were syntenic with each other across the breakpoints.

Finally, due to the inherent noise in linkage-disequilibrium based estimates of ρ, the lack of evidence for a synteny break in Panu_3.0 is not positive evidence that the Panu_3.0 assembly is correct.

Inference of crossovers in a baboon pedigree

We utilized a previously described vcf file for the baboons shown in Figure 6 which was mapped using Panu_2.0 coordinates and lifted over to Panu_3.0 coordinates. We considered only biallelic SNPs, and required a depth ≥ 15, QUAL > 50 and genotype quality (GQ) ≥ 40 in order to make a genotype call. We further required an allelic balance (AB) of > 0.3 for heterozygote calls and AB < 0.07 for homozygote calls, and excluded all repetitive regions as described in [48].

We focused our analyses on those SNPs that were most informative about recent crossover events. For example, to detect paternal crossovers, we restricted our analyses to SNPs where 10173 was heterozygous, both 9841 and 12242 were homozygous, and all 9 offspring had genotype calls. (For maternal crossovers, we required 10173 to be homozygous and both 9841 and 12242 to be heterozygous.) For these sites, it is straightforward to infer which allele (coded as 0 for reference allele and 1 for alternative allele) was passed on from 10173 to his offspring. While the haplotypic phase of 10173 is unknown, we can infer crossover events based on the minimum number of crossovers needed to be consistent with the observed patterns of inheritance in the offspring of 10173 ([49]). For example, Figure 5c shows that the inheritance pattern near position 29.38 requires at least 3 crossovers (e.g., in individuals 17199, 18385 and 19348).

For each potential error in the Panu_3.0 assembly, we converted the breakpoint location into Panu_2.0 coordinates and verified synteny between Panu_2.0 and Panu_3.0 across the breakpoint region. We then determined whether there were an abnormally large number of crossovers inferred right at the breakpoint. Specifically, if we inferred at least 3 crossover events (out of 18 total meioses, 9 paternal and 9 maternal), then we considered this as evidence that the Panu_3.0 assembly is incorrect, as in Figure 5c (cf. ‘Linkage Support’ column in Table 2). Note that the converse isn’t true: fewer than 3 inferred crossover events is not evidence that the Panu_3.0 assembly is correct at a particular location.

Repeat Analysis

We analyzed the repeat content of the Panubis1.0 and Panu_3.0 genome assemblies using RepeatMasker [50] version open-4.0.8 in sensitive mode and was run with blastp version 2.0MP-WashU using the RepeatMasker Combined Database: Dfam_Consensus-20181026, RepBase-20181026. The following parameters were used to run RepeatMasker: RepeatMasker -engine wublast -species 'papio anubis' -s -no_is -cutoff 255 -frag 20000

Figure S6 summarizes the distribution of various types of repeats found in the two genome assemblies. We found that the genome assemblies are comparable in terms of their repeat content.

Conclusion

The development and commercialization of new technologies by companies such as Illumina, 10x Genomics, Bionano Genomics, Dovetail Genomics and Phase Genomics has enabled researchers to cheaply generate fully *de novo* genome assemblies with high scaffold contiguity (e.g., [40]; [41]; [33]; [36]; [42]). When used in combination with long-read sequences (e.g., from Oxford Nanopore or Pacific Biosciences), these technologies can produce high-quality genome assemblies at a fraction of the cost of traditional clone library based approaches (e.g., [41]; [43]). In this context, our assembly Panubis1.0 provides a 10-fold increase in contig N50 size and a 240-fold increase in scaffold N50 size relative to Panu_3.0 at less than 1% of the reagent cost. The contiguity of this assembly will be especially useful for future studies where knowing the genomic location is important (e.g., hybridization or recombination studies).

One natural question that arises with any new genome assembly is how one assesses that an assembly is *'correct'*. Indeed, some of the recently published Hi-C based assemblies have not provided any corroborating evidence supporting their assemblies (e.g., [44]). Here, we used three independent sources of information to provide evidence that 11 out of 12 large syntenic differences identified from the dotplots are correct in our new baboon assembly (Panubis1.0) relative to the previous assembly Panu_3.0 (Table 2). In all, the incorporation of optical maps, linkage and linkage disequilibrium data provide substantially more support for our assembly than was produced by previous Hi-C based assemblies (e.g., [41]; [42]; [43]), and counters any potential criticism of the fact that our genome assembly (using individual '15944' from the SNPRC baboon colony) comes from a different individual from the previous baboon assembly (individual 1X1155 from the SNPRC baboon colony).

There is however a larger list of 21 inversion differences between Panubis1.0 and Panu_3.0 where the Hi-C data do not provide definitive evidence on which orientation is correct (Table 3). While Hi-C based assemblies may be prone to small contig inversions within scaffolds, this should be less of a problem for the large inversions

outlined here since there will be few interactions that span the full length of the contig, and the correct orientation is generally apparent from the higher weight of links [16]. These changes to the baboon assembly should be considered provisional, until additional data can be collected (e.g., high-coverage long read data) that provides a more definitive answer.

Availability of supporting data

All of the raw sequence data from individual 15944, as well as the Panubis1.0 assembly are available without restriction from NCBI under BioProject PRJNA527874. New RNA-seq data used for genome annotation are available under BioProject PRJNA559725. The genome annotation report and raw files can be found at https://www.ncbi.nlm.nih.gov/genome/annotation_euk/Papio_anubis/104/.

Competing interests

The authors declare that they have no competing interests.

Acknowledgements

We thank Jenny Tung for providing some of the high-molecular weight DNA used in this study.

Funding

The work was supported in part by NIH grants R24 OD017859 (to LAC and JDW), R01 GM115433 (to JDW), R01 GM094402 (to YSS), R35 GM134922 (to YSS), R01 HG005946 (to PYK) and by a Packard Fellowship for Science and Engineering (to YSS). YSS is a Chan Zuckerberg Biohub Investigator.

Author contributions

JDW, LAC and YSS conceived the project. JG, SD, SS, MLS and PYK generated data for the project. MLS and SSB performed the genome assembly. SSB, MLS, JR, TPV and JDW performed the other analyses. SSB and JDW wrote the manuscript with contributions from all authors.

References

1. VandeBerg JL, Williams-Blangero S, Tardif SD, editors. The baboon in biomedical research. Springer Science & Business Media; 2009 Jun 4.

2. McGill HC, McMahan CA, Kruski AW, Kelley JL, Mott GE. Responses of serum lipoproteins to dietary cholesterol and type of fat in the baboon. *Arteriosclerosis*. 1981;1:337–44.

3. Kushwaha RS, Reardon CA, Lewis DS, Qi Y, Rice KS, Getz GS, et al. Effect of dietary lipids on plasma activity and hepatic mRNA levels of cholesteryl ester transfer protein in high- and low-responding baboons (Papio species). *Metabolism*. 1994;43:1006–12.

4. Singh AT, Rainwater DL, Kammerer CM, Sharp RM, Poushesh M, Shelledy WR, et al. Dietary and genetic effects on LDL size measures in baboons. *Arterioscler Thromb Vasc Biol.* 1996;16:1448–53.

5. Kammerer CM, Rainwater DL, Cox LA, Schneider JL, Mahaney MC, Rogers J, et al. Locus controlling LDL cholesterol response to dietary cholesterol is on baboon homologue of human chromosome 6. *Arterioscler Thromb Vasc Biol.* 2002;22:1720–5.

6. Rainwater DL, Kammerer CM, Mahaney MC, Rogers J, Cox LA, Schneider JL, et al. Localization of genes that control LDL size fractions in baboons. *Atherosclerosis.* 2003;168:15–22.

7. Martin LJ, Blangero J, Rogers J, Mahaney MC, Hixson JE, Carey KD, et al. A quantitative trait locus influencing activin-to-estrogen ratio in pedigreed baboons maps to a region homologous to human chromosome 19. *Hum Biol.* 2001;73:787–800.

8. Sherwood RJ, Duren DL, Havill LM, Rogers J, Cox LA, Towne B, et al. A genomewide linkage scan for quantitative trait loci influencing the craniofacial complex in baboons (Papio hamadryas spp.). *Genetics.* 2008;180:619–28.

9. Havill LM, Mahaney MC, Cox LA, Morin PA, Joslyn G, Rogers J. A quantitative trait locus for normal variation in forearm bone mineral density in pedigreed baboons maps to the ortholog of human chromosome 11q. *J Clin Endocrinol Metab.* 2005;90:3638–45.

10. Havill LM, Cox LA, Rogers J, Mahaney MC. Cross-species replication of a serum osteocalcin quantitative trait locus on human chromosome 16q in pedigreed baboons. *Calcif Tissue Int.* 2005;77:205–11.

11. Rainwater DL, Cox LA, Rogers J, VandeBerg JL, Mahaney MC. Localization of multiple pleiotropic genes for lipoprotein metabolism in baboons. *J Lipid Res.* 2009;50:1420–8.

12. Cox LA, Glenn J, Ascher S, Birnbaum S, VandeBerg JL. Integration of genetic and genomic methods for identification of genes and gene variants encoding QTLs in the nonhuman primate. *Methods.* 2009;49:63–9.

13. Telenti A, Pierce LCT, Biggs WH, di Iulio J, Wong EHM, Fabani MM, et al. Deep sequencing of 10,000 human genomes. *Proc Natl Acad Sci USA.* 2016;113:11901–6.

14. McCarthy S, Das S, Kretzschmar W, Delaneau O, Wood AR, Teumer A, et al. A reference panel of 64,976 haplotypes for genotype imputation. *Nat Genet.* 2016;48:1279–83.

15. Halldorsson BV, Palsson G, Stefansson OA, Jonsson H, Hardarson MT, Eggertsson HP, Gunnarsson B, Oddsson A, Halldorsson GH, Zink F, Gudjonsson SA. Characterizing mutagenic effects of recombination through a sequence-level genetic map. Science. 2019 Jan 25;363(6425):eaau1043.

16. Karczewski KJ, Francioli LC, Tiao G, Cummings BB, Alföldi J, Wang Q, Collins RL, Laricchia KM, Ganna A, Birnbaum DP, Gauthier LD. Variation across 141,456 human exomes and genomes reveals the spectrum of loss-of-function intolerance across human protein-coding genes. BioRxiv. 2019 Jan 1:531210.

17. NHLBI Trans-Omics for Precision Medicine. https://www.nhlbiwgs.org/. Accessed 10 Jan 2019.

18. Lander ES, Linton LM, Birren B, Nusbaum C, Zody MC, Baldwin J, et al. Initial sequencing and analysis of the human genome. *Nature.* 2001;409:860–921.

19. International Human Genome Sequencing Consortium. Finishing the euchromatic sequence of the human genome. *Nature*. 2004;431:931–45.

20. International HapMap Consortium. A haplotype map of the human genome. *Nature.* 2005;437:1299–320.

21. International HapMap Consortium. A second generation human haplotype map of over 3.1 million SNPs. Nature. 2007 Oct;449(7164):851.

22. International HapMap 3 Consortium, Altshuler DM, Gibbs RA, Peltonen L, Altshuler DM, Gibbs RA, et al. Integrating common and rare genetic variation in diverse human populations. *Nature*. 2010;467:52–8.

23. 1000 Genomes Project Consortium, Abecasis GR, Altshuler D, Auton A, Brooks LD, Durbin RM, et al. A map of human genome variation from population-scale sequencing. *Nature.* 2010;467:1061–73.

24. 1000 Genomes Project Consortium, Abecasis GR, Auton A, Brooks LD, DePristo MA, Durbin RM, et al. An integrated map of genetic variation from 1,092 human genomes. *Nature*. 2012;491:56–65.

25. 1000 Genomes Project Consortium, Auton A, Brooks LD, Durbin RM, Garrison EP, Kang HM, et al. A global reference for human genetic variation. *Nature*. 2015;526:68–74.

26. Wall JD, Schlebusch SA, Alberts SC, Cox LA, Snyder-Mackler N, Nevonen KA, et al. Genomewide ancestry and divergence patterns from low-coverage sequencing data reveal a complex history of admixture in wild baboons. *Mol Ecol*. 2016;25:3469–83.

27. Rogers J, Raveendran M, Harris RA, Mailund T, Leppälä K, Athanasiadis G, Schierup MH, Cheng J, Munch K, Walker JA, Konkel MK. The comparative genomics and complex population history of Papio baboons. Science advances. 2019 Jan 1;5(1):eaau6947.

28. Weisenfeld NI, Kumar V, Shah P, Church DM, Jaffe DB. Direct determination of diploid genome sequences. *Genome Res*. 2017;27:757–67.

29. Ma ZS, Li L, Ye C, Peng M, Zhang YP. Hybrid assembly of ultra-long Nanopore reads augmented with 10x-Genomics contigs: Demonstrated with a human genome. Genomics. 2019 Dec 1;111(6):1896-901.

30. Qin M, Wu S, Li A, Zhao F, Feng H, Ding L, Ruan J. LRScaf: improving draft genomes using long noisy reads. BMC genomics. 2019 Dec 1;20(1):955.

31. Xu GC, Xu TJ, Zhu R, Zhang Y, Li SQ, Wang HW, Li JT. LR_Gapcloser: a tiling path-based gap closer that uses long reads to complete genome assembly. GigaScience. 2019 Jan;8(1):giy157.

32. Walker BJ, Abeel T, Shea T, Priest M, Abouelliel A, Sakthikumar S, Cuomo CA, Zeng Q, Wortman J, Young SK, Earl AM. Pilon: an integrated tool for comprehensive microbial variant detection and genome assembly improvement. PloS one. 2014;9(11).

33. Dudchenko O, Batra SS, Omer AD, Nyquist SK, Hoeger M, Durand NC, et al. De novo assembly of the Aedes aegypti genome using Hi-C yields chromosome-length scaffolds. *Science*. 2017;356:92–5.

34. Phase Genomics. https://www.phasegenomics.com/. Accessed 10 Jan 2019.

35. Dudchenko O, Shamim MS, Batra S, Durand NC, Musial NT, Mostofa R, Pham M, St Hilaire BG, Yao W, Stamenova E, Hoeger M. The Juicebox Assembly Tools module facilitates de novo assembly of mammalian genomes with chromosome-length scaffolds for under $1000. Biorxiv. 2018 Jan 1:254797.

36. Matthews, Benjamin J., et al. Improved reference genome of Aedes aegypti informs arbovirus vector control. *Nature*. 2018;563:501–7.

37. Simão FA, Waterhouse RM, Ioannidis P, Kriventseva EV, Zdobnov EM. BUSCO: assessing genome assembly and annotation completeness with single-copy orthologs. *Bioinformatics.* 2015;31:3210–2.

38. Perelman P, Johnson WE, Roos C, Seuánez HN, Horvath JE, Moreira MA, Kessing B, Pontius J, Roelke M, Rumpler Y, Schneider MP. A molecular phylogeny of living primates. PLoS Genet. 2011 Mar 17;7(3):e1001342.

39. Skov L, Schierup MH, Danish Pan Genome Consortium. Analysis of 62 hybrid assembled human Y chromosomes exposes rapid structural changes and high rates of gene conversion. PLoS genetics. 2017 Aug 28;13(8):e1006834.

40. Mostovoy Y, Levy-Sakin M, Lam J, Lam ET, Hastie AR, Marks P, et al. A hybrid approach for de novo human genome sequence assembly and phasing. *Nat Methods.* 2016;13:587–90.

41. Bickhart DM, Rosen BD, Koren S, Sayre BL, Hastie AR, Chan S, et al. Single-molecule sequencing and chromatin conformation capture enable de novo reference assembly of the domestic goat genome. *Nat Genet.* 2017;49:643–50.

42. Rice, Edward S., et al. Improved genome assembly of American alligator genome reveals conserved architecture of estrogen signaling. *Genome Research.* 2017;27:686–696.

43. Kalbfleisch TS, Rice ES, DePriest MS, Walenz BP, Hestand MS, Vermeesch JR, Brendan LO, Fiddes IT, Vershinina AO, Saremi NF, Petersen JL. Improved reference genome for the domestic horse increases assembly contiguity and composition. Communications biology. 2018 Nov 16;1(1):1-8.

44. Nuss AB, Sharma A, Gulia-Nuss M. Chicago and Dovetail Hi-C proximity ligation yield chromosome length scaffolds of Ixodes scapularis genome. bioRxiv. 2018 Jan 1:392126.

45. Jain M, Koren S, Miga KH, Quick J, Rand AC, Sasani TA, et al. Nanopore sequencing and assembly of a human genome with ultra-long reads. *Nat Biotechnol.* 2018;36:338–45.

46. Rao, Suhas SP, et al. A 3D map of the human genome at kilobase resolution reveals principles of chromatin looping. *Cell.* 2014;159:1665–1680.

47. Chan AH, Jenkins PA, Song YS. Genome-wide fine-scale recombination rate variation in Drosophila melanogaster. PLoS genetics. 2012 Dec;8(12).

48. Robinson, Jacqueline A., et al. Analysis of 100 high-coverage genomes from a pedigreed captive baboon colony. *Genome Research.* 2019;29:848–856.

49. Coop G, Wen X, Ober C, Pritchard JK, Przeworski M. High-resolution mapping of crossovers reveals extensive variation in fine-scale recombination patterns among humans. *Science*. 2008;319:1395–8.

50. Tarailo-Graovac M, Chen N. Using RepeatMasker to identify repetitive elements in genomic sequences. Current protocols in bioinformatics. 2009 Mar;25(1):4-10.

Figure 1. Illustration of our genome assembly strategy.

Figure 2. The Hi-C map of our Panubis1.0 genome. The figure represents the Hi-C map obtained by aligning Hi-C paired-end reads to the Panubis1.0 genome assembly laid out on the X-axis as well as the Y-axis. Since each read-pair consists of two reads, a position (*i, j*) on this map represents the number of read-pairs where one read aligned to position *i* and the other read aligned to position *j* on the Panubis1.0 genome. The intensity of each pixel in this Hi-C map represents the number of reads aligning within that bin. The Hi-C map has been drawn at the resolution of 1.25 megabases. Each blue square on the diagonal represents a chromosome-length scaffold. Autosomes are listed first, ordered by size, and the last square corresponds to the X chromosome. The axes are labelled in units of megabases.

Figure 3. Dotplots showing chromosome Y synteny suggest that the Panubis1.0 chromosome Y is putatively at least a part of the true chromosome Y. A dotplot between rhesus chromosome Y and Panubis1.0 putative chromosome Y is shown on the left, while a dotplot between the chimpanzee chromosome Y and the human chromosome Y is shown on the right. The axes labels are in units of megabases. The phylogenetic distance between baboon and rhesus macaque is similar to that between human and chimpanzee. Hence, the broadly conserved synteny between the rhesus and baboon putative chromosome Y as compared to the synteny between the chimp and human chromosome Y, suggests that the scaffold representing the putative chromosome Y in the Panubis1.0 assembly is indeed capturing at least a large part of chromosome Y.

Figure 4. Dotplots showing alignment of Panu_3.0 reference-assisted chromosomes vs. Panubis1.0 chromosome-length scaffolds. The Panu_3.0 assembly is shown on the Y-axis and the Panubis1.0 assembly is shown on the X-axis. Each dot represents the position of a syntenic block between the two assemblies as determined by the nucmer alignment. The color of the dot reflects the orientation of the individual alignments (purple indicates consistent orientation and blue indicates inconsistent orientation). The dotplots illustrate that there are chromosomes containing large inversions and translocations in the Panu_3.0 assembly with respect to the Panubis1.0 assembly.

Figure 5. Evidence for misassembly on chromosome NC_018167.2 in Panu_3.0. **a)** Bionano optical map alignment to the Panu_3.0 assembly demonstrates there is an inversion on chromosome NC_018167.2 beginning at ~29.38 Mb and ending at ~44.71 Mb. **b)** Estimates of the population recombination rate ρ near the potential synteny breaks of the inversion identified on chromosome NC_018167.2. **c)** Shown on the x-axis is positions along chromosome NC_018167.2 in Panu_3.0 where each row represents one of the 9 offsprings of sire 10173. Switches between red and blue within

a row represent a recombination event. The two vertical black lines represent locations where three or more recombinations occur at the same locus indicating a potential misassembly.

Figure 6. Pedigree of baboons used in linkage analysis.

Assembly	10X	10X contigs	10X contigs + Nanopore scaffolding	10X contigs + Nanopore Scaffolding + Nanopore gap filling	10X contigs + Nanopore scaffolding + Nanopore gap filling + Illumina polishing	Panubis1.0	Panu_3.0
Total Length of Scaffolds	2,892,554,220	2,809,352,255	2,871,292,557	2,871,210,925	2,870,847,162	2,869,821,163	2,959,373,024
Number of Scaffolds	24,513	87,632	15,803	15,803	15,803	11,145	63,235
Scaffold N50	15,720,195	84,258	1,695,573	1,695,772	1,695,642	140,274,886	585,721
Total Gap Length	83,203,960	0	50,344,034	2,030,908	2,030,908	2,321,983	22,434,732
Total Length of Contigs	2,809,350,260	2,809,352,255	2,820,948,523	2,869,180,017	2,868,816,254	2,867,510,325	2,937,001,527
Number of Contigs	87,347	87,632	62,252	17,004	17,004	15,243	122,216
Contig N50	84,258	84,258	134,222	1,469,760	1,469,602	1,455,705	138,819

Table 1. Assembly statistics for each step of the adopted assembly strategy. Total Length of Scaffolds is the sum of lengths of scaffolds (including A, C, G, T and N) in each scaffold. Total Gap Length is the total number of N's in the assembly. Contigs are constructed by splitting the assembly at every stretch of at least one N. The total length of contigs is the sum of the number of sequenced base pairs (including only A, C, G and T) in each scaffold.

Panu_3.0 chromosome	Panu_3.0 Start	Panu_3.0 End	Panu_2.0 Start	Panu_2.0 End	Type	Linkage support	BNG support	LDhelmet support
NC_018164.2	88.05	104.99	87.61	104.98	Inv	start[1]	yes	unknown[1]
NC_018167.2	29.38	44.71	29.25	44.53	Inv	start + end	yes	start + end
NC_018156.2	4.04	8.67	4.18	8.63	Inv	no	yes[2]	no
NC_018162.2	82.42	86.47	81.91	84.01	Trans	start + end	no[3]	no
NC_018166.2	104.28	108.05	103.66	107.44	Inv	no	yes	no
NC_018165.2	15.93	19.48	15.85	19.40	Inv	no	no	no
NC_018166.2	96.94	100.12	96.39	99.54	Trans	start + end	yes[4]	start + end
NC_018160.2	36.05	36.75	35.88	36.55	Trans	no	yes[4]	start
NC_018163.2	23.19	23.66	0	0.47	Trans	no	yes[2]	no
NC_018164.2	4.05	4.49	3.99	4.45	Trans	no[5]	yes	no
NC_018165.2	100.91	101.18	100.31	100.59	Trans	no	yes	no
NC_018152.2	166.73	166.89	169.86	170.10	Trans	start + end	yes	end

Table 2. Likely large (>100 Kb) assembly errors in Panu_3.0, ordered by size. Start and end positions in Panu_3.0 and Panu_2.0 are in units of mega bases (MB). Note that a 'no' in the 'Linkage support' or 'LDhelmet support' columns is inconclusive, and should not be interpreted as support for the Panu_3.0 assembly being correct.

[1] Unable to determine whether linkage and LDhelmet provide support at the end breakpoint due to a lack of synteny between Panu_2.0 and Panu_3.0
[2] Panu_2.0 assembly appears to be correct
[3] BNG maps do not support a translocation with these breakpoints. However, they do support a potential large SV at the starting breakpoint
[4] BNG maps support the presence of a large SV, which may be a translocation
[5] Linkage data suggests a potential polymorphic inversion (in 16413) partially overlapping with this interval

Panubis1.0 chromosome	Panubis1.0 Start (Mb)	Panubis1.0 End (Mb)	Panu_3.0 chromosome	Panu_3.0 chromosome	Panu_3.0 Start (Mb)	Panu_3.0 End (Mb)
NC_044992.1	28.89	45.01	CM001506.2	NC_018167.2	29.38	44.79
NC_044995.1	0	13	CM001509.2	NC_018170.2	0	13.31
NC_044987.1	101.26	106.48	CM001504.2	NC_018165.2	101.44	107.53
NC_044978.1	176.83	181.37	CM001495.2	NC_018156.2	175.08	180.09
NC_044986.1	86.61	90.73	CM001499.2	NC_018160.2	85.56	90.3
NC_044988.1	0	3.5	CM001505.2	NC_018166.2	0	3.78
NC_044996.1	86.67	89.58	CM001511.2	NC_018172.2	86.91	90.23
NC_044982.1	154.35	156.82	CM001497.2	NC_018158.2	155.71	158.53
NC_044984.1	7.96	10.58	CM001501.2	NC_018162.2	8.03	10.83
NC_044991.1	33.09	35.09	CM001500.2	NC_018161.2	32.46	35.05
NC_044996.1	93.67	95.52	CM001511.2	NC_018172.2	94.22	96.59
NC_044981.1	68.61	71.05	CM001494.2	NC_018155.2	69.37	71.65
NC_044996.1	40.49	42.78	CM001511.2	NC_018172.2	41.15	43.34
NC_044996.1	10.01	11.79	CM001511.2	NC_018172.2	10.2	12.06
NC_044996.1	31.8	33.37	CM001511.2	NC_018172.2	32.11	33.97
NC_044979.1	142.32	144.05	CM001493.2	NC_018154.2	141.96	143.71
NC_044996.1	90.77	92.54	CM001511.2	NC_018172.2	91.42	92.99
NC_044993.1	63.59	65.52	CM001510.2	NC_018171.2	62.31	63.73
NC_044991.1	26.79	28.49	CM001500.2	NC_018161.2	26.52	27.82
NC_044980.1	0.02	0.78	CM001496.2	NC_018157.2	0.02	1.26
NC_044979.1	0	0.73	CM001493.2	NC_018154.2	0	0.75

Table 3. Additional large (>100 Kb) inversion differences between Panubis1.0 and Panu_3.0, ordered by size We cannot definitively determine which orientation is correct for the following inversions, and they should be considered as provisional.

Figure 1

Click here to access/download;Figure;Figure_1.pdf

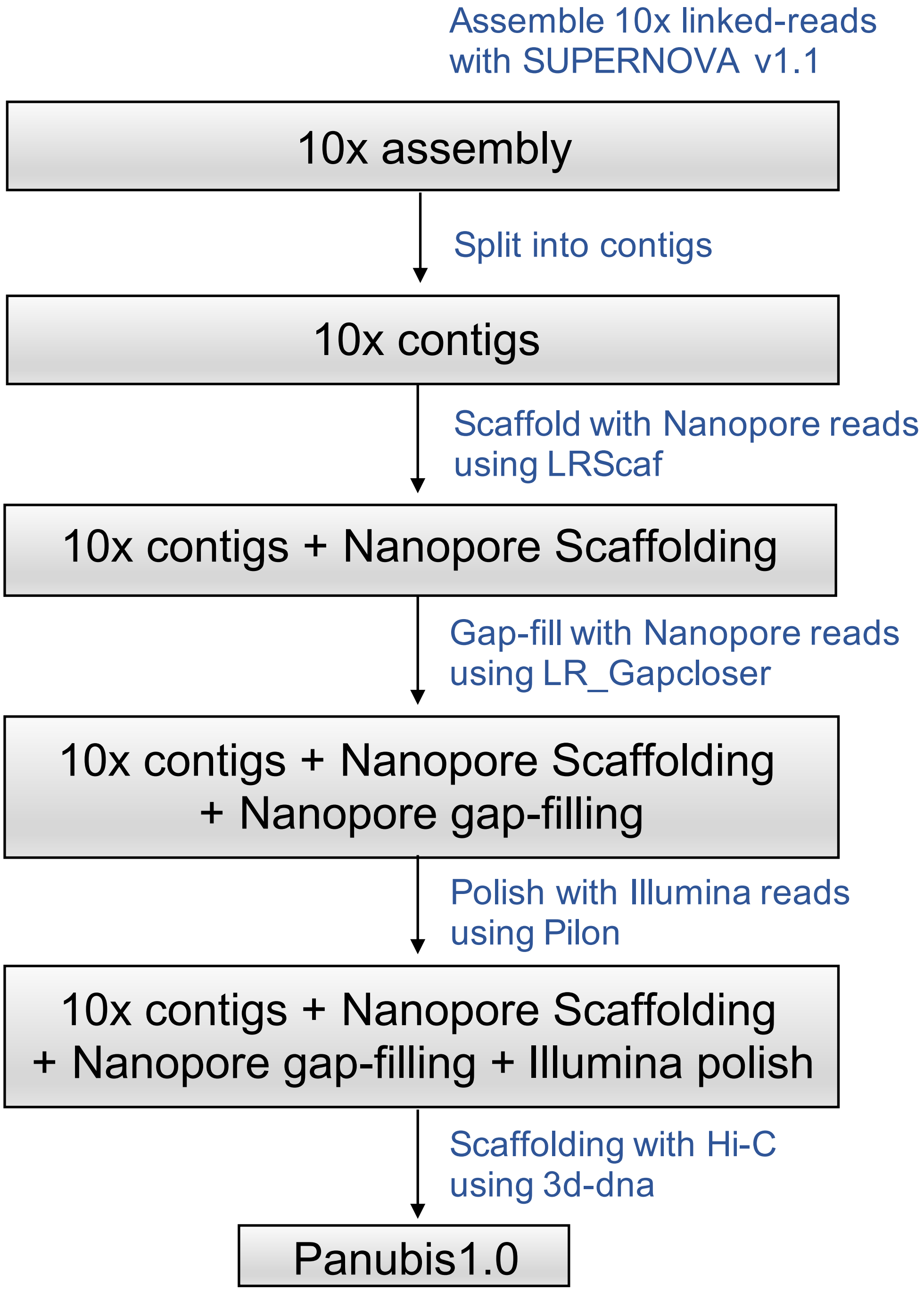

Figure 2

Click here to access/download;Figure;Figure_2.pdf

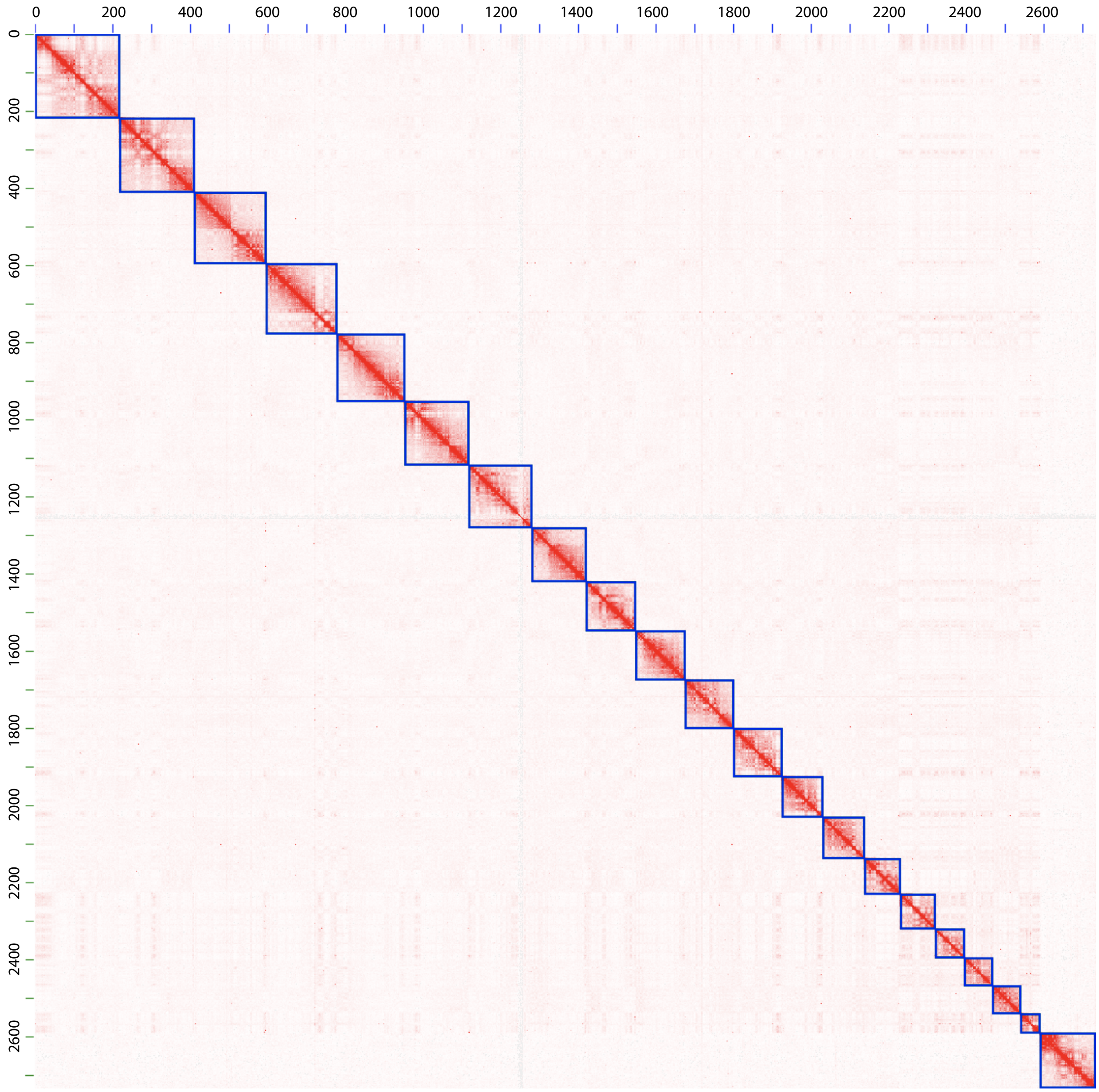

Figure 3

Click here to access/download;Figure;Figure_3.pdf

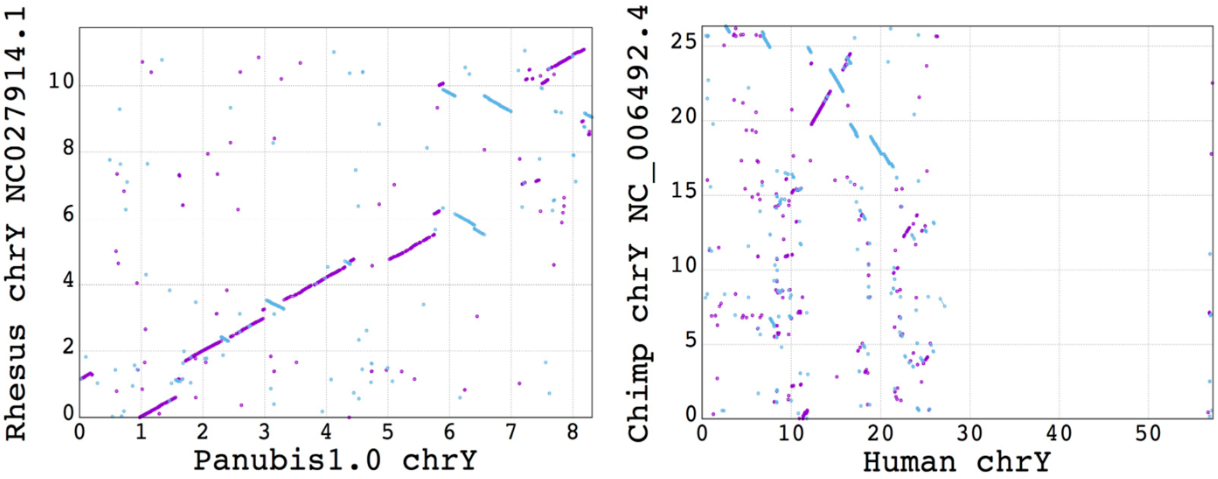

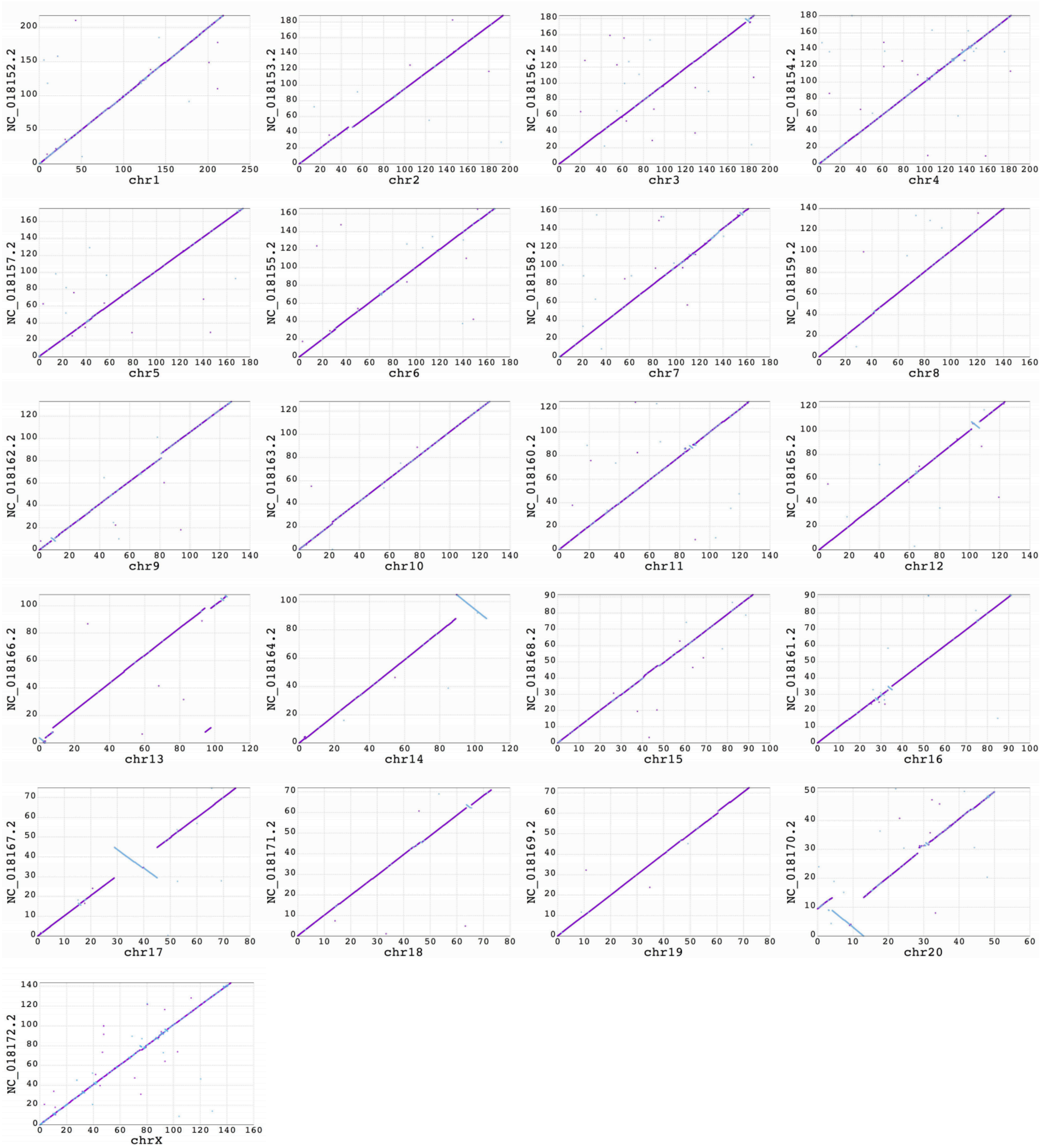
NC_018152.2
chr1
NC_018153.2
chr2
NC_018156.2
chr3
NC_018154.2
chr4
NC_018157.2
chr5
NC_018155.2
chr6
NC_018158.2
chr7
NC_018159.2
chr8
NC_018162.2
chr9
NC_018163.2
chr10
NC_018160.2
chr11
NC_018165.2
chr12
NC_018166.2
chr13
NC_018164.2
chr14
NC_018168.2
chr15
NC_018161.2
chr16
NC_018167.2
chr17
NC_018171.2
chr18
NC_018169.2
chr19
NC_018170.2
chr20
NC_018172.2
chrX

Figure 5

Click here to access/download;Figure;Figure_5.pdf

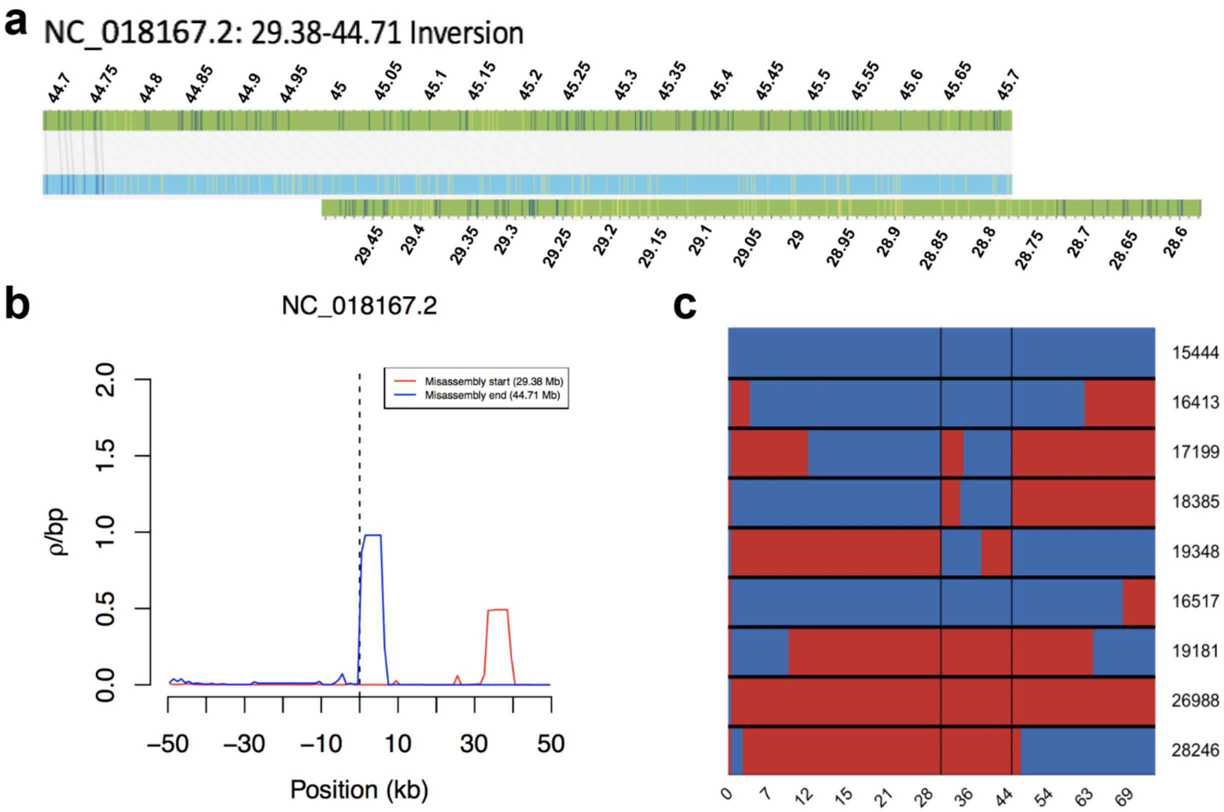

Figure 6

Click here to access/download;Figure;Figure_6.pdf

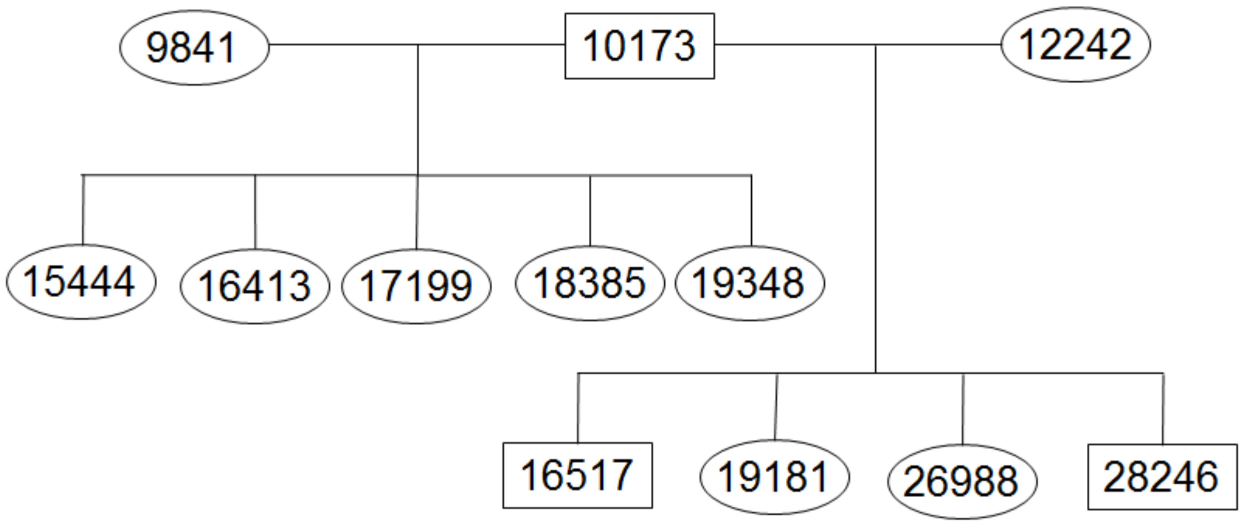

Click here to access/download
Supplementary Material
FINAL_SupplementaryFigures.pdf
