# Supplementary material for: Accurate assembly of the olive baboon (Papio anubis) genome using long-read and Hi-C data
Source: Gigascience. 2020 Dec 7;9(12):giaa134. doi: 10.1093/gigascience/giaa134 (PMC7719865; doi:10.1093/gigascience/giaa134)
Supplement: giaa134_Supplemental_File [file giaa134_supplemental_file.pdf]

Supplementary Information

Accurate assembly of the olive baboon (*Papio anubis*) genome using long-read and Hi-C data

Sanjit Singh Batra[1], Michal Levy-Sakin[2], Jacqueline Robinson[3], Joseph Guillory[4], Steffen Durinck[4,5], Tauras P. Vilgalys[6], Pui-Yan Kwok[2,3], Laura A. Cox[7,8], Somasekar Seshagiri[4], Yun S. Song[1,9,10] and Jeffrey D. Wall[3,*]

[1]Computer Science Division, University of California, Berkeley, CA 94720;
[2]Cardiovascular Research Institute, University of California San Francisco, San Francisco, CA 94143;
[3]Institute for Human Genetics, University of California San Francisco, San Francisco, CA 94143;
[4]Department of Molecular Biology, Genentech, Inc., 1 DNA Way, South San Francisco, CA 94080;
[5]Bioinformatics and Computational Biology Department, Genentech, Inc., 1 DNA Way, South San Francisco, CA 94080;
[6]Department of Evolutionary Anthropology, Duke University, Durham, NC 27705
[7]Center for Precision Medicine, Department of Internal Medicine, Section of Molecular Medicine, Wake Forest School of Medicine, Winston-Salem, NC 27101
[8]Southwest National Primate Research Center, Texas Biomedical Research Institute, San Antonio, TX 78245
[9]Department of Statistics, University of California, Berkeley, CA 94720;
[10]Chan Zuckerberg Biohub, San Francisco, CA 94158

*Corresponding Author: Jeff.Wall@ucsf.edu

This document contains Supplementary Figures S1-S6.

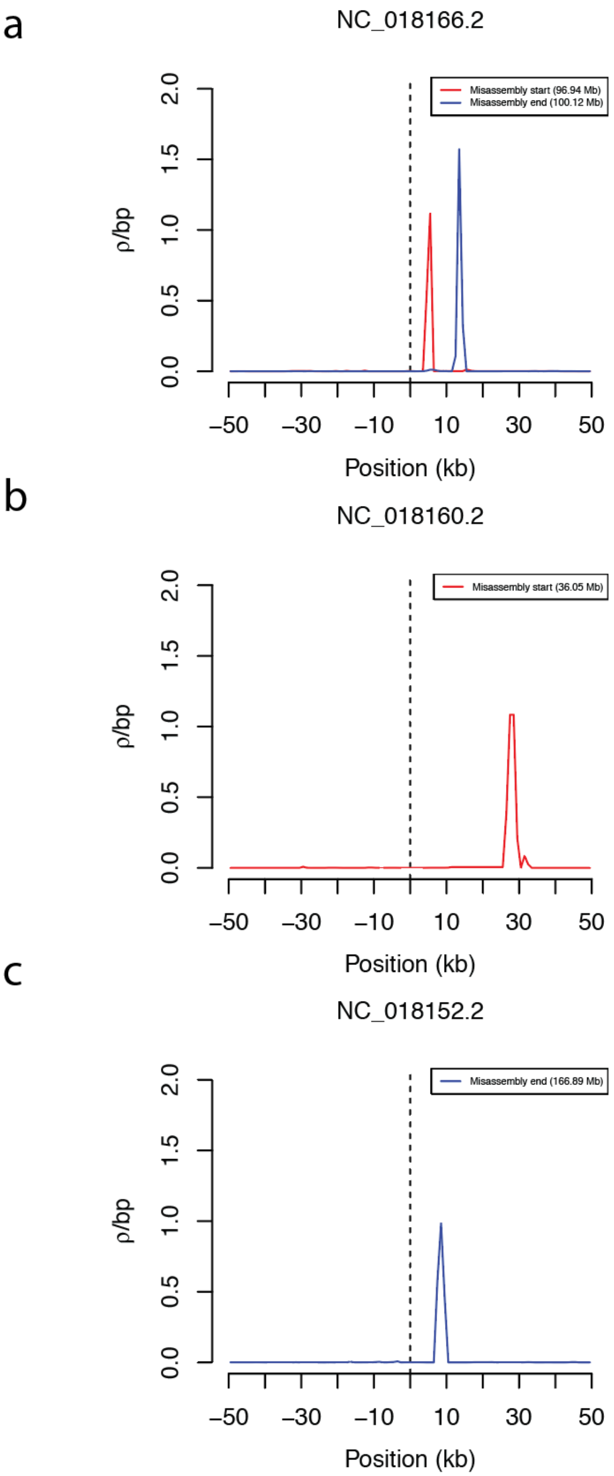

Figure S1: Linkage disequilibrium based evidence for misassemblies in Panu_3.0. Estimates of the population recombination rate ρ near the potential synteny breaks of the misassemblies identified in chromosomes a) NC_018166.2 b) NC_018160.2 and c) NC_018152.2. Red represents the beginning of a misassembly event and blue represents the end of a misassembly event.

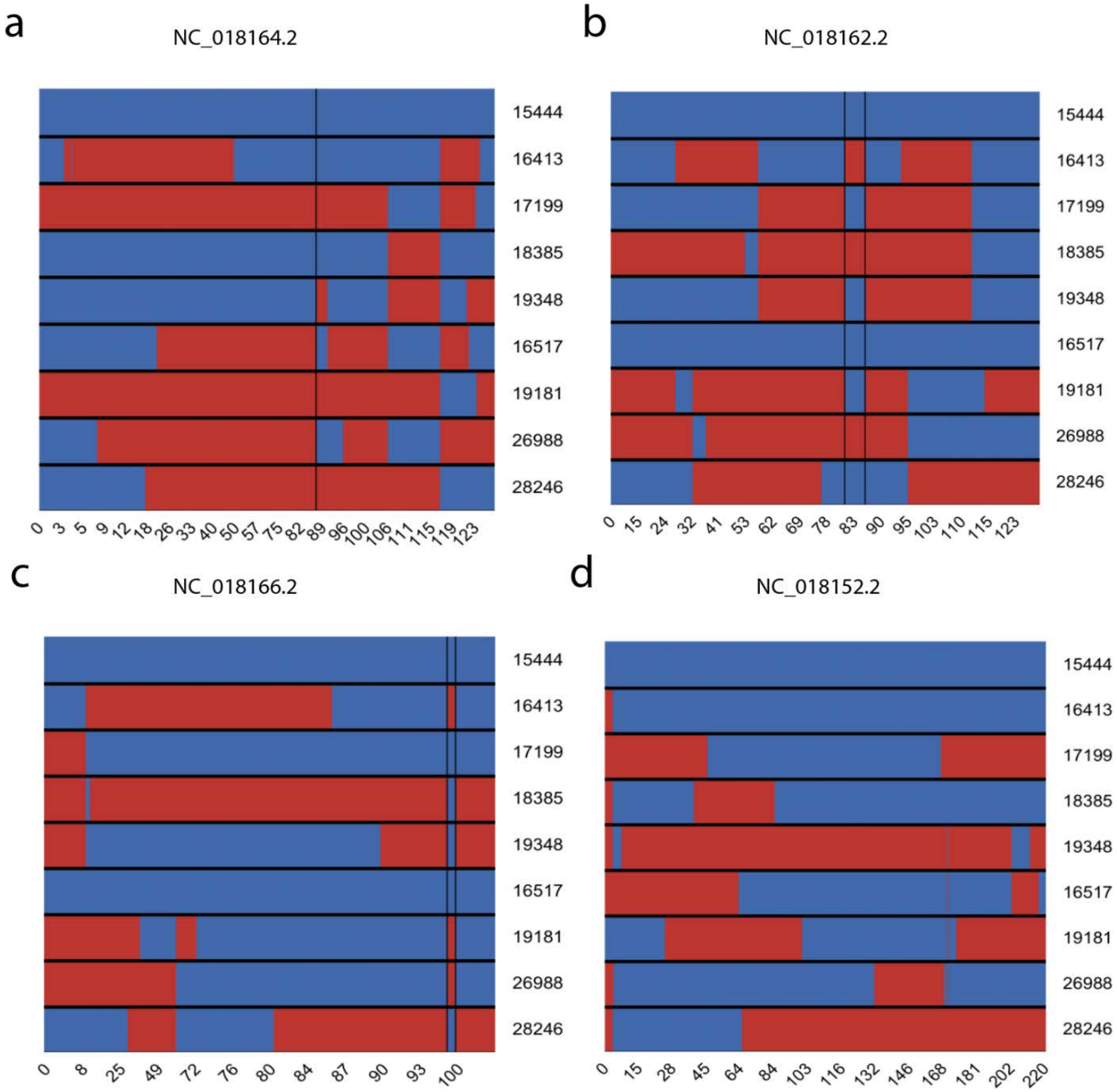

Figure S2: Recombination based evidence for misassemblies in Panu_3.0.
Shown on the x-axis are positions along chromosomes in Panu_3.0 where each row represents one of the 9 offsprings of sire 10173. Switches between red and blue within a row represent a recombination event. The vertical black lines represent locations where three or more recombinations occur at the same locus indicating a potential misassembly; except in (d) where recombination occurs at ~167Mb but is not shown by a vertical black line.

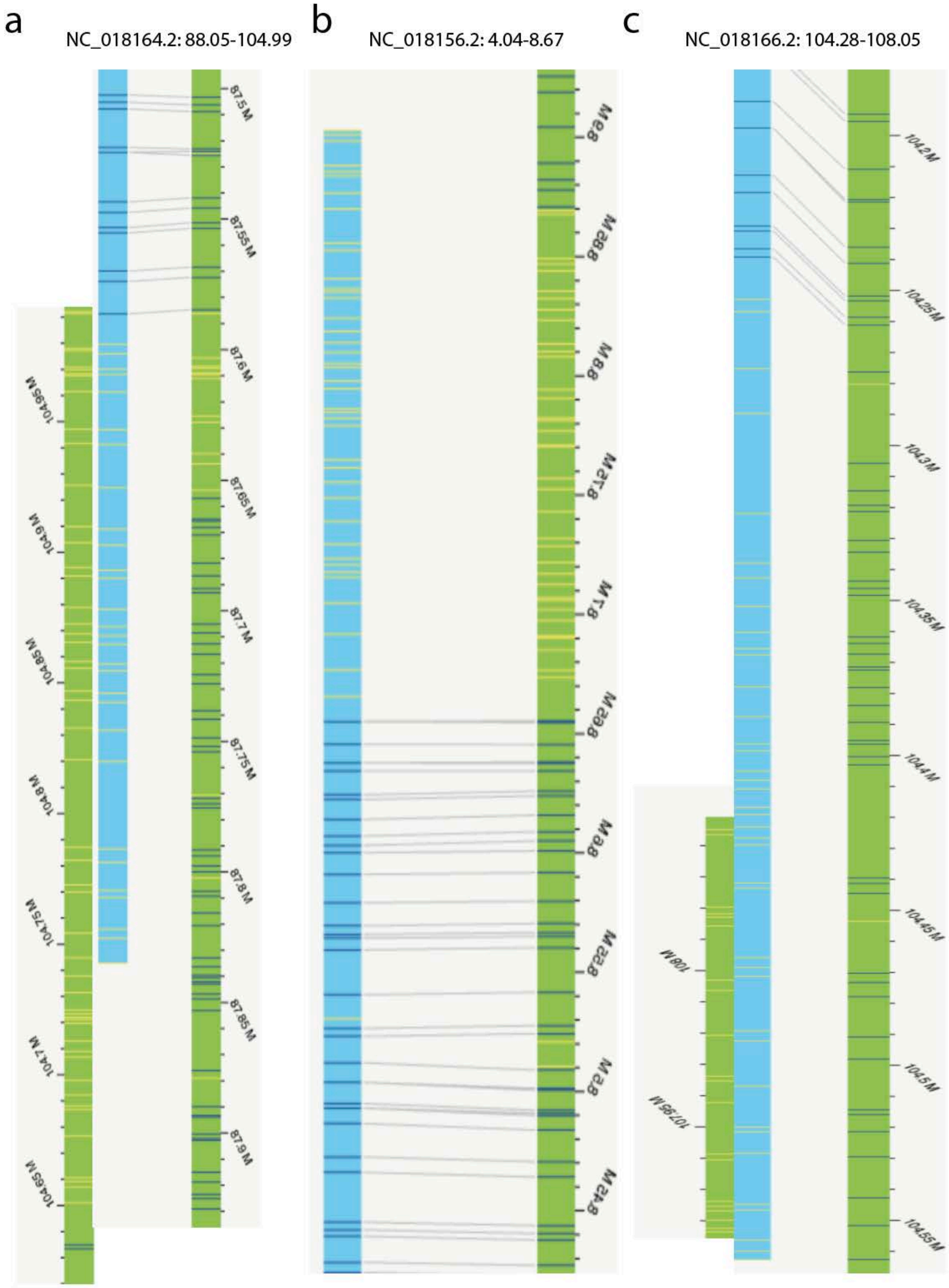

Figure S3: Evidence for inversions in Panu_3.0 based on bionano alignment.
a) Inversion on chromosome NC_018164.2 demonstrated by bionano optical map alignment.
b) Alignment to bionano optical map shows inverted coordinates due to an inversion on chromosome NC_018156.2.
c) Bionano optical map alignment shows an inversion on chromosome NC_018166.2.

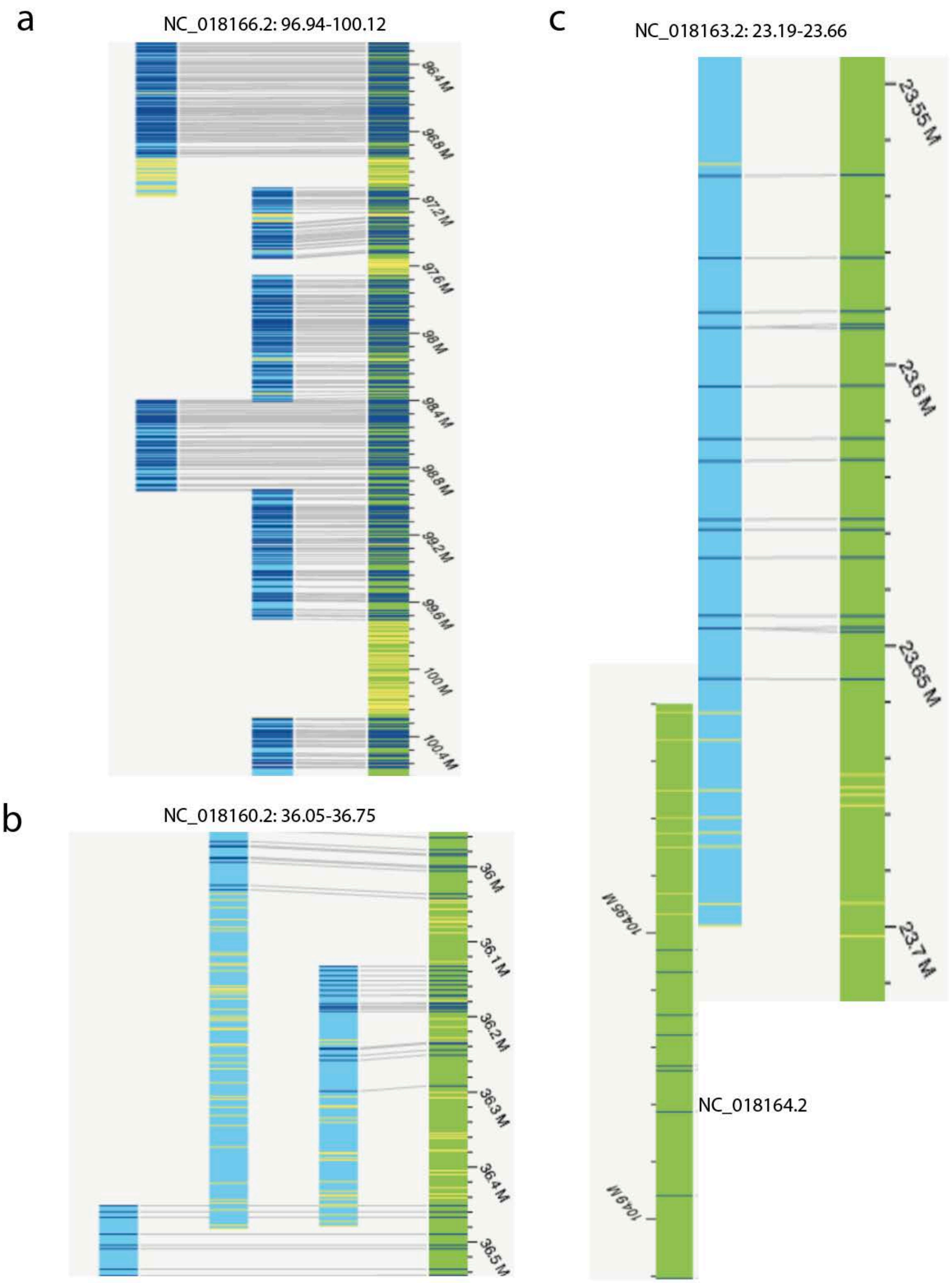

Figure S4: Evidence for translocations in Panu_3.0 based on bionano alignment.
a) Breaks in bionano alignment on chromosome NC_018166.2 indicate a misassembly.
b) Bionano optical map alignment demonstrate a misassembly on chromosome NC_018160.2.
c) Bionano optical map alignment shows a translocation between chromosomes NC_018163.2 and NC_018164.2.

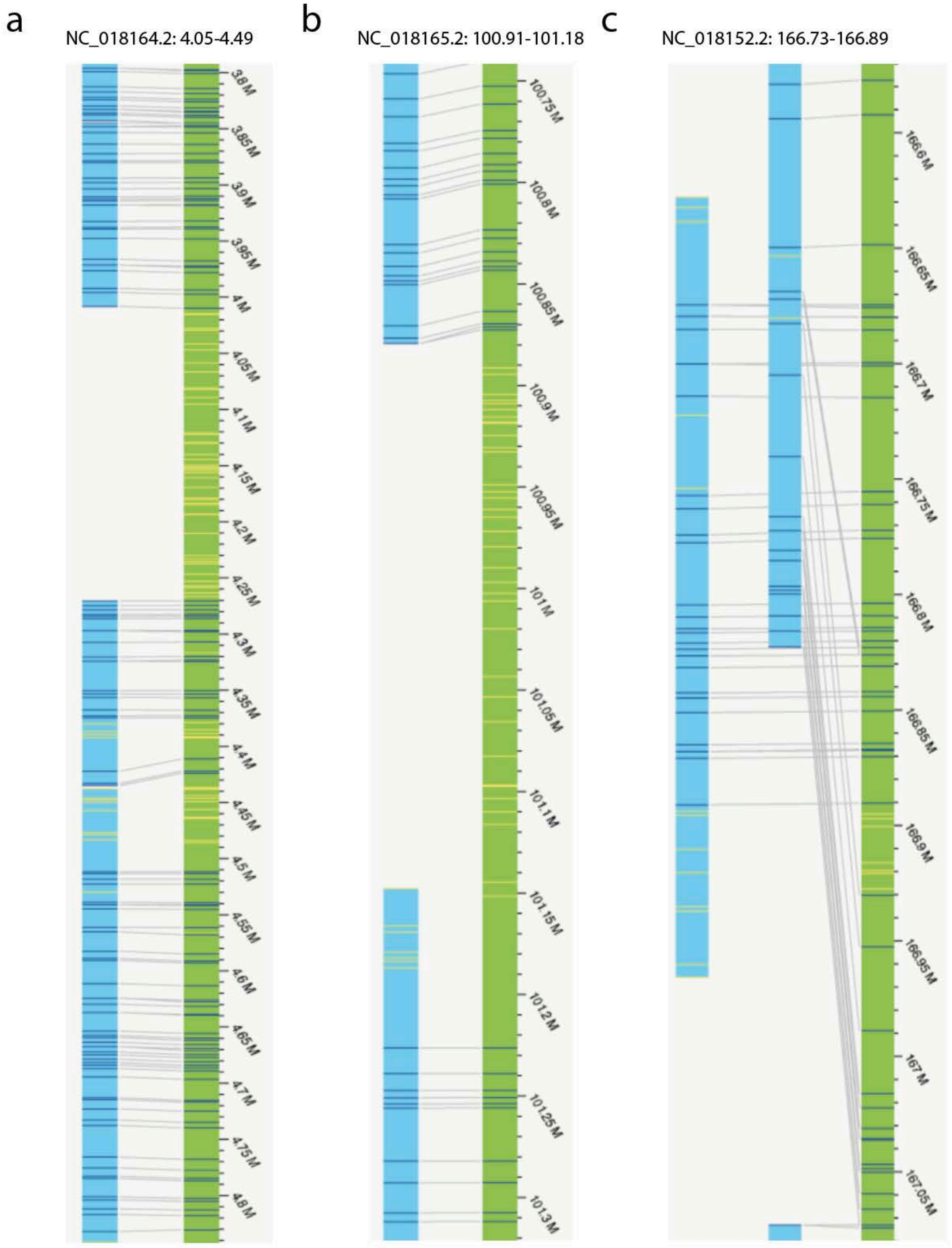

Figure S5: Evidence for translocations in Panu_3.0 based on bionano alignment.
a) Breaks in bionano alignment on chromosome NC_018164.2 indicate a misassembly.
b) Bionano optical map alignment demonstrate a misassembly on chromosome NC_018165.2.
c) Bionano optical map alignment shows a translocation on chromosome NC_018152.2.

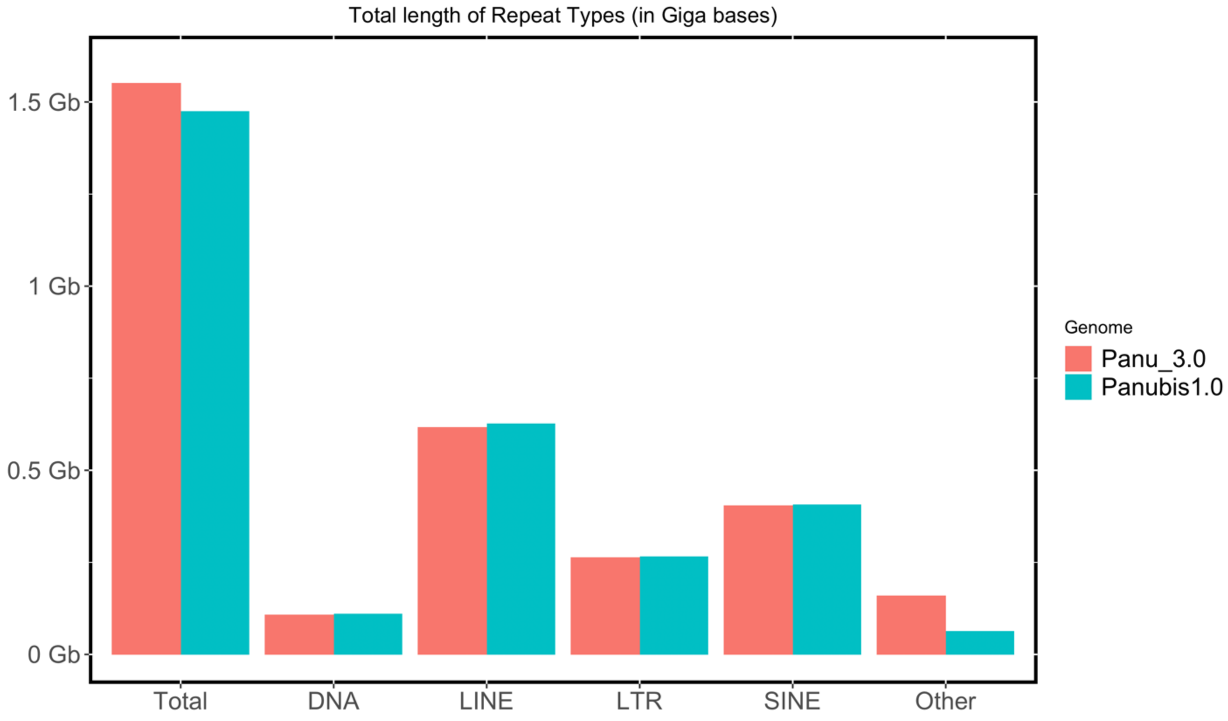

Figure S6: RepeatMasker output analysis for Panubis1.0 and Panu_3.0.
Total length, represented in Giga bases (Gb), of different repeat types in Panubis1.0 and Panu_3.0 obtained by analyzing the genomes with RepeatMasker.
