# Supplementary material for: Accurate assembly of the olive baboon (Papio anubis) genome using long-read and Hi-C data
Source: Gigascience. 2020 Dec 7;9(12):giaa134. doi: 10.1093/gigascience/giaa134 (PMC7719865; doi:10.1093/gigascience/giaa134)
Supplement: giaa134_Response_to_Reviewer_Comments_Original_Submission [file giaa134_response_to_reviewer_comments_original_submission.pdf]

Author's Response To Reviewer Comments

Close

We thank both reviewers for their helpful comments, and believe our manuscript has improved with their feedback. We provide a point-by-point discussion of their concerns below.

Reviewer #1:

- Reference style is not identical, examinations are needed.

We have corrected the reference style to reflect the instructions provided to authors on the GigaScience website.

- Both package versions and parameter settings of the software used should be given.

We have updated the text to include the package versions and parameter settings of software that have been used.

- In my view, during the assembly process, the Nanopore long-reads should be polished by Illumina paired-end reads firstly.

We communicated with the author of LR_Gapcloser, which is the tool we used to scaffold the contigs with nanopore long reads.

We asked them the following question:
"Would you recommend using raw nanopore reads or error-corrected nanopore reads. If you suggest error-corrected nanopore reads, would you suggest self-correction or correction with illumina reads?

The author responded with:
"——Directly use the raw reads...We found that using the uncorrected read has better performance than using the corrected reads."

Consequently, we used the raw Nanopore long-reads.

- The figure legend of the Hi-C heatmap need to be improved, some details are missing.

We have modified the figure legend of the Hi-C heatmap to provide additional details.

- I can not understand the meaning of the Fig.3. What is the reader supposed to conclude from the figure?

We have modified the figure legend for Figure 3 to describe the motivation behind it. In particular, we wanted to demonstrate why we believe that the scaffold labeled chromosome Y by us, is putatively at least a part of the true chromosome Y of the genome, by showing that chromosome Y synteny between Panubis1.0 and rhesus is similar to the chromosome Y synteny between human and chimp.

- The genome completeness assessment shows that the completeness of Panu_3.0 is better than that of Panubis1.0 (93.4% Vs 93%), please explain this.

We performed an updated BUSCO analysis using the Euarchontoglires gene set instead of the broader Mammalia gene set provided by BUSCO. We have updated the manuscript to show that the number of

“Complete” genes found in Panubis1.0 and Panu_3.0 is almost identical.

- It would be good for the authors to explain how they have addressed the main problem with the HiC for the accurate orientation of the inversions within the scaffolds.

We thank the reviewer for their comment. We have added an explanation of how we have addressed the main problem with Hi-C data of the accuracy of orientation of short contigs, within the discussion section. In particular, since our contig N50 is > 1 megabase and the orientation accuracy of contigs with Hi-C data increases with contig length, this should not be a big problem for our dataset.

Reviewer #2:

There are some issues with the presentation of data in this version of the paper, as some of the data is missing (repeats) not presented (ONT), and some should be clarified (BUSCO scores, Tables 1 and 2).

We have tried our best to incorporate these suggestions into the manuscript.

- 1. This investigation would greatly benefit from description of the repeats. Isn't is the whole point of using long read data?

We have included an analysis of repeats for Panubis1.0 as well as Panu_3.0 using the RepeatMasker software.

- 2. BUSCO score is lower in their final assembly. This is strange, and needs to be addressed somewhere.

We have added a refined analysis of the BUSCO scores to the manuscript using the Euarchontoglires gene set instead of the broader Mammalia gene set provided by BUSCO. We have updated the manuscript to show that the number of “Complete” genes found in Panubis1.0 and Panu_3.0 is almost identical.

- 3. While the assembly has benefited from the 15x ONT coverage, i was not able to find information on the reads. The authors used the ONT data to assemble scaffolds with LR_Scaf (published as recently as last December) and calculated gap lengths. In LRScaf paper - the authors mention that the benefits of their method is speed and resource consumption. Therefore, it is not clear why there were not used for assembly, only for scaffolding with no justification given.

The Canu assembler documentation (which can be found at https://canu.readthedocs.io/en/latest/quick-start.html) recommends that "For eukaryotic genomes, coverage more than 20x is enough to outperform current hybrid methods, however, between 30x and 60x coverage is the recommended minimum." Since we only had 15x nanopore reads, we consequently didn't attempt to assemble the nanopore reads de novo and opted to use them for scaffolding of contigs instead. We have also modified the discussion section to provide this reasoning.

- 4. Table 1 and 2 should be combined, just add the last column of Table 2 to the end of table 1, there is no reason to report the Panubis 1.0 statistics twice.

We have modified Table 1 and Table 2 and combined them into a single table.

- 5. Finally, I am a little concerned with continuous classification of animals according to their biomedical need, rather with their evolutionary and ecological significance. This trend is recently prevalent in GigaScience as well as the other journals and reflect an extreme anthropocentric view. I think that the paper will benefit from a statement on how this data contributes to the completeness of the primate research, evolutionary, comparative and conservation studies.

We completely agree with this sentiment and have modified the text to highlight the long-standing importance of baboons as models for non-medical studies (such as evolutionary genetics and animal behavior).

Close
