# Supplementary material for: Accurate assembly of the olive baboon (Papio anubis) genome using long-read and Hi-C data
Source: Gigascience. 2020 Dec 7;9(12):giaa134. doi: 10.1093/gigascience/giaa134 (PMC7719865; doi:10.1093/gigascience/giaa134)
Supplement: giaa134_Response_to_Reviewer_Comments_Revision_1 [file giaa134_response_to_reviewer_comments_revision_1.pdf]

Author's Response To Reviewer Comments

Close

We thank the reviewer for their comments. We provide our response to their suggestions below.

Reviewer #1:

- The description of the repeats is still very simple. Despite that total length of repeats by RepeatMasker are present in supplementary figure, this is not enough. Much details are missing, do they perform this based on homology or de novo? maybe both. From their results, the repeats for Panubis1.0 is not higher even lower that Panu_3.0. This is strange, please make some new efforts to provide more evidences. I do not know if the de novo method will work but the authors should try it.

The repeat analysis was done by running RepeatMasker version open-4.0.8 in sensitive mode and was run with blastp version 2.0MP-WashU using the RepeatMasker Combined Database: Dfam_Consensus-20181026, RepBase-20181026. The following parameters were used to run RepeatMasker: RepeatMasker -engine wublast -species 'papio anubis' -s -no_is -cutoff 255 -frag 20000

We have also included these details into the manuscript.

- The authors used the Euarchontoglires gene set instead of the broader Mammalia gene set provided by BUSCO. However, the result is not very good. At least, no new evidence to show the high quality of their genome. I do not know if this are related with their assembly strategy. I am wondering if the 15x ONT coverage could be used for assembly directly, it is hard to estimate from the sequence coverage. But the authors should think about this carefully and some more work needed to be done to fix this problem.

The Canu assembler documentation (which can be found at https://canu.readthedocs.io/en/latest/quick-start.html) recommends that "For eukaryotic genomes, coverage more than 20x is enough to outperform current hybrid methods, however, between 30x and 60x coverage is the recommended minimum." Since we only had 15x nanopore reads, we consequently didn't attempt to assemble the nanopore reads de novo and opted to use them for scaffolding of contigs instead. This reasoning is outlined in the discussion section.

Close
