# Supplementary material for: Accurate assembly of the olive baboon (Papio anubis) genome using long-read and Hi-C data
Source: Gigascience. 2020 Dec 7;9(12):giaa134. doi: 10.1093/gigascience/giaa134 (PMC7719865; doi:10.1093/gigascience/giaa134)
Supplement: giaa134_Response_to_Reviewer_Comments_Revision_2 [file giaa134_response_to_reviewer_comments_revision_2.pdf]

Author's Response To Reviewer Comments

Close

We have made the requested changes to the manuscript.

Close
