# Supplementary material for: Accurate assembly of the olive baboon (Papio anubis) genome using long-read and Hi-C data
Source: Gigascience. 2020 Dec 7;9(12):giaa134. doi: 10.1093/gigascience/giaa134 (PMC7719865; doi:10.1093/gigascience/giaa134)
Supplement: giaa134_Reviewer_2_Report_Original_Submission — Taras K Oleksyk, Ph.D. -- 4/10/2020 Reviewed [file giaa134_reviewer_2_report_original_submission.pdf]

Reviewer Report

Title: Accurate assembly of the olive baboon (Papio anubis) genome using long-read and Hi-C data

Version: Original Submission **Date:** 4/10/2020

Reviewer name: Taras K Oleksyk, Ph.D.

Reviewer Comments to Author:

This paper brings forward a de novo genome genome of the olive baboon assembled using 10x, ONT (Nanopore), HiC and BioNano maps corrected for the large assemble errors with Panu_3.0, leading to an improved genome annotation. This data is valuable as the previous baboon assembly based on the Illumina short reads and mate pairs was a lot more fragmented. A nice feature was to infer the recombination rate and annotating the crossover breakpoints. The resulting genome reaches 2.87 Gb of continuous sequence with less than 0.1% of uncalled bases (gaps), and a 10 times improved N50 containing 93 complete genes according to BUSCO
There are some issues with the presentation of date in this version of the paper, as some of the data is missing (repeats) not presented (ONT), and some should be clarified (BUSCO scores, Tables 1 and 2).
1. This investigation would greatly benefit from description of the repeats. Isn't is the whole point of using long read data?
2. BUSCO score is lower in their final assembly. This is strange, and needs to be addressed somewhere.
3. While the assembly has benefited from the 15x ONT coverage, i was not able to find information on the reads. The authors used the ONT data to assemble scaffolds with LR_Scaf (published as recently as last December) and calculated gap lengths. In LRScaf paper - the authors mention that the benefits of their method is speed and resource consumption. Therefore, it is not clear why there were not used for assembly, only for scaffolding with no justification given.
4. Table 1 and 2 should be combined, just add the last column of Table 2 to the end of table 1, there is no reason to report the Panubis 1.0 statistics twice.
5. Finally, I am a little concerned with continuous classification of animals according to their biomedical need, rather with their evolutionary and ecological significance. This trend is recently prevalent in GigaScience as well as the other journals and reflect an extreme anthropocentric view. I think that the paper will benefit from a statement on how this data contributes to the completeness of the primate research, evolutionary, comparative and conservation studies.

Level of Interest

Please indicate how interesting you found the manuscript: Choose an item.

Quality of Written English

Please indicate the quality of language in the manuscript: Choose an item.

Declaration of Competing Interests

Please complete a declaration of competing interests, considering the following questions:

- Have you in the past five years received reimbursements, fees, funding, or salary from an organisation that may in any way gain or lose financially from the publication of this manuscript, either now or in the future?
- Do you hold any stocks or shares in an organisation that may in any way gain or lose financially from the publication of this manuscript, either now or in the future?
- Do you hold or are you currently applying for any patents relating to the content of the manuscript?
- Have you received reimbursements, fees, funding, or salary from an organization that holds or has applied for patents relating to the content of the manuscript?
- Do you have any other financial competing interests?
- Do you have any non-financial competing interests in relation to this paper?

If you can answer no to all of the above, write 'I declare that I have no competing interests' below. If your reply is yes to any, please give details below.

I declare that I have no competing interests

I agree to the open peer review policy of the journal. I understand that my name will be included on my report to the authors and, if the manuscript is accepted for publication, my named report including any attachments I upload will be posted on the website along with the authors' responses. I agree for my report to be made available under an Open Access Creative Commons CC-BY license (http://creativecommons.org/licenses/by/4.0/). I understand that any comments which I do not wish to be included in my named report can be included as confidential comments to the editors, which will not be published.

Choose an item.

To further support our reviewers, we have joined with Publons, where you can gain additional credit to further highlight your hard work (see: https://publons.com/journal/530/gigascience). On publication of this paper, your review will be automatically added to Publons, you can then choose whether or not to claim your Publons credit. I understand this statement.

Yes Choose an item.
